# Transcriptome and Neuroendocrinome Responses to Environmental Stress in the Model and Pest Insect *Spodoptera frugiperda*

**DOI:** 10.3390/ijms26020691

**Published:** 2025-01-15

**Authors:** Wei Gong, Jan Lubawy, Paweł Marciniak, Guy Smagghe, Małgorzata Słocińska, Dongdong Liu, Tongxian Liu, Shunhua Gui

**Affiliations:** 1 Guizhou Provincial Key Laboratory for Agricultural Pest Management of the Mountainous Region, Institute of Entomology, Guizhou University, Guiyang 550025, China; 15564806356@163.com (W.G.); liudd@gzu.edu.cn (D.L.); tx.liu@gzu.edu.cn (T.L.); 2 Department of Animal Physiology and Developmental Biology, Faculty of Biology, Institute of Experimental Biology, Adam Mickiewicz University in Poznań, 61-0614 Poznań, Poland; jan.lubawy@amu.edu.pl (J.L.); pmarcin@amu.edu.pl (P.M.); malgorzata.slocinska@amu.edu.pl (M.S.); 3 Cellular and Molecular Life Sciences, Department of Biology, Vrije Universiteit Brussel (VUB), 1050 Brussels, Belgium; 4 Institute of Plant Health and Medicine, Guizhou University, Guiyang 550025, China

**Keywords:** fall armyworm, brain, transcriptome, neuropeptides, neuropeptide receptors, environmental constraint

## Abstract

The fall armyworm, *Spodoptera frugiperda*, is one of the most notorious pest insects, causing damage to more than 350 plant species, and is feared worldwide as an invasive pest species since it exhibits high adaptivity against environmental stress. Here, we therefore investigated its transcriptome responses to four different types of stresses, namely cold, heat, no water and no food. We used brain samples as our interest was in the neuroendocrine responses, while previous studies used whole bodies of larvae or moths. In general, the responses were complex and encompassed a vast array of neuropeptides (NPs) and biogenic amines (BAs). The NPs were mainly involved in ion homeostasis regulation (ITP and ITPL) and metabolic pathways (AKH, ILP), and this was accompanied by changes in BA (DA, OA) biosynthesis. Cold and no-water stress changed the NP gene expression with the same patterns of expression but clearly separated from each other, and the most divergent pattern of expression was shown after no-food stress. In conclusion, our data provide a foundation in an important model and pest insect with candidate NPs and BAs and other marker candidate genes in response to environmental stress, and also potential new targets to manage pest insects.

## 1. Introduction

Generally, insects have a short life cycle and are powerful and rapidly adaptive organisms. They are the largest group in the Arthropoda phylum and are capable of surviving in a large variety of environments [1,2,3]. In thermoregulation, insects are different than mammals since they are poikilotherm organisms, also called ectotherms, as their body heat is derived exclusively from their external environments, affecting metabolism, growth, development and behavior [4,5].

In the brains of insects, the neurosecretory cells (NSCs) synthesize and release an array of neuropeptides/hormones, which are chief regulators to carry out many life activities and mechanisms in insects and adaptation to stress conditions [6]. For instance, significant hormones can regulate their growth and development with cuticle formation, digestion, excretion and molting. Some good examples are ion transport peptide (ITP), inotocin (ITC), diuretic hormones (DH31 and DH44), calcitonin (CAL), neuropeptide F (NPF) and allatostatin-B, which are involved in the response to cold stress, while the capability peptides (CAPA) respond to both cold and dry stresses and the adipokinetic hormone (AKH) affects starvation stress. Corazonin, insulin-like peptides (ILPs), tachykinin (TK), neuropeptide F (NPF) and other neuropeptides (NPs) in regulating carbohydrates and lipoproteins are involved in the environmental stress of insects [1,7].

On thermal sensitivity, different investigations have revealed that in *Drosophila melanogaster*, thermosensitive neurons in the antennae respond to 0.5 °C [8], and in each antenna, there are three heat receptor and three cold receptor neurons [8]. The response of these thermoreceptor neurons is dependent on the fluctuating concentration change of essential ions Mg^2+^, K^+^, Na^+^ and Ca^2+^, and these ions’ movements across the membrane are temperature dependent [9]. This ion flow promotes the release of chemical messengers, including neuropeptides (NPs) (e.g., proctolin, allatostatin, allatotropin, prothoracicotropic hormone (PTTH) and AKH)), biogenic amines (BAs) (e.g., serotonin, epinephrine, octopamine (OA), histamine, dopamine (DA), norepinephrine and tyramine (TY)), acetylcholine neurotransmitter and amino acids (glutamate and gamma-aminobutyric acid (GABA)). These chemical messengers play a role as neurotransmitters and neurohormones, which further interact with other neurons to develop different physiological and behavioral changes in response to the temperature stress.

A well-studied example is the exposure to high temperatures in *Drosophila* [5]. Upon exposure to heat stress, NPs, BAs and heat shock proteins (HSPs) produce a stress response in the body that serves as protection of the nervous system and other body organs from variant heat damage. An increase in temperature leads to higher metabolic rates in the insect, and the smaller body size helps the insect to reduce its metabolic rate to survive. The *corpora allata* (CA) and prothoracic glands (PGs) release high levels of juvenile hormone (JH) and 20-hydroxyecdysone, respectively, due to temperature rise in the hemolymph, regulating the condition of diapause and various other metabolic and behavioral processes. Also, the insect under heat stress can mediate the thermoregulation process by increasing evaporative water loss via spiracles, changing the functions of flight muscles and color change of the body by cuticular pigment, and changing their position according to heat stress and moving to cooler habitats.

For this project, we have chosen to work with the fall armyworm, *Spodoptera frugiperda* (J.E. Smith) (Lepidoptera: Noctuidae). It is one of the world’s most harmful pest insects; it is distributed worldwide in different climate zones and has a strong adaptive potential to thermal and dry stresses [10]. Originally, this pest species was native to tropical and subtropical regions of North and South America. But with its strong migration ability and adaptability, *S. frugiperda* has spread and invaded many countries around the world, covering different temperature zones and botanical compositions. Beyond the Americas, it has invaded Africa, Asia and Australia and is also moving towards Europe. Its habitat is quite complex and it can survive on a wide range of hosts (353 plant species) [11]. Such good adaptive abilities, as well as the invasiveness of this insect, have attracted the attention of many researchers not only at the ecological level but also at the molecular level, with previous papers on *S. frugiperda* transcriptomics in response to environmental stresses confirming the interest in this species [12,13,14,15,16,17,18,19,20].

Our objectives were to record and analyze the responses to different types of stresses in *S. frugiperda*. Particularly, we investigated the responses of the brain since the NSCs are chief regulators to provide an appropriate strategy to fight stressful events. Here, it should be noted that in our study, we used brain samples as our interest had a focus on the neuroendocrine responses, while previous studies used whole bodies of larvae or moths. We used RNA sequencing technology and analyzed the brains of *S. frugiperda* larvae that were briefly exposed to low (4 °C) and high temperature (42 °C) compared to normal temperature (25 °C). In addition, considering that the larvae of *S. frugiperda* can survive in a complex habitat and feed a wide range of host plants, we investigated their response to no-food and no-water stress. We believe that with our results, we can provide a foundation in an important model and pest insect, for which we now have available molecular tools, including a complete genome and an efficient protocol for CRISPR-Cas9 [21]. They can indicate candidate NPs and BAs in response to environmental stress, as well as marker candidate genes of *S. frugiperda* in response to environmental stress and potential new insecticide targets.

## 2. Results

### 2.1. RNA-Sequencing Data

We constructed 15 RNA libraries using the Illumina HiSeq platform. Per stress condition and the control, we performed three biological replicates. After filtering low-quality reads, a total of 164.24 Gb of clean data was obtained from the 15 samples, and the clean data of each sample reached 9.53 Gb. The Q30 base percentage was 94.37% and above. The number of clean reads and clean bases was from 31,884,413 to 41,152,362 and 9,526,556,450 to 12,299,637,872, respectively. The GC content of all sample clean reads ranged between 47.10% and 51.28% (Table 1). The clean reads of each sample were sequenced against the designated reference genome (GenBank number: GCA_023101765.3, GCA_012979215.2, GCA_019297735.2, GCA_015832365.1, GCA_026413635.1, GCA_900240015.1, GCA_002811805.1, GCA_000753635.2, GCA_002213285.2, GCA_011064685.2) separately and the efficiency of the comparison ranged from 82.71% to 85.33%.

### 2.2. Analysis of DEGs Under Stress Conditions

**Figure 1:**
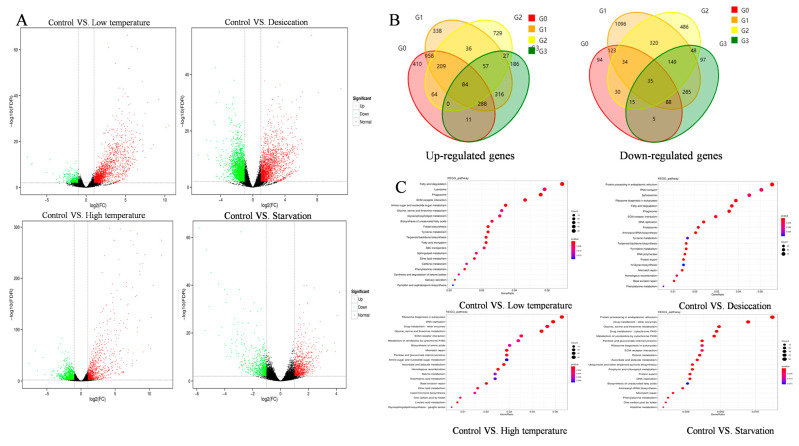
(**A**) Volcano plot of the DEGs in the brain of *S. frugiperda* under four different stress conditions. (**B**) Venn diagram of the number of DEGs (up- and downregulated genes). Note: G0 represents Control vs. Cold (4 °C), G1 represents Control vs. No-water, G2 represents Control vs. Heat (42 °C), G3 represents Control vs. No-food. (**C**) Pathway enrichment statistics for all transcripts mRNA under different environmental stresses.

The DEGs under stress were identified by comparing the expression levels of transcripts under the different stress conditions and the control (fold change ≥ 2.0 and *p* < 0.01) (Figure 1A and Table 2). Cold stress caused 2448 DEGs, with 2024 genes that were upregulated genes and 424 downregulated compared to the control. With heat stress, there were 2323 DEGs with 1206 upregulated and 1117 downregulated ones. No-water stress showed 4416 DEGs with 2286 genes upregulated and 2130 downregulated. While no-food stress resulted in 1691 DEGs, including 969 upregulated and 722 downregulated genes compared to the control. A total of 35 common genes were downregulated and 84 upregulated under the four different stress conditions (Figure 1B).

#### 2.2.1. Top-50 DEGs After Cold Stress

After cold stress, out of analyzed genes, cholesterol 7-desaturase and JH epoxide hydrolase-like showed complete inhibition in expression (100%). The data revealed four isoforms of cytochrome P450, which were downregulated by 10 to 41 times. Interestingly, two genes encoding immune-associated proteins (inducible metalloproteinase inhibitor protein-like and protein NDNF-like) were downregulated by 48 and 18 times, respectively. The rest of the genes were suppressed from 90% to 99%. The five most upregulated genes were putative fatty acyl-CoA reductase, chitin deacetylase 8-like, cuticle protein 8-like and ecdysone oxidase, whose expression increased by 620-, 282-, 191- and 180-fold, respectively (Supplementary Table S1). One gene, mucin-5AC, was not detectable in the control, while its expression increased during cold stress. The rest of the top upregulated genes increased from 63-fold (alpha-tocopherol transfer protein-like) to 300-fold (uncharacterized LOC118271331).

#### 2.2.2. Top 50 DEGs After Heat Stress

During heat stress, three genes, namely chitin deacetylase 1, uncharacterized oxidoreductase YrbE-like and spore coat protein T-like, were completely downregulated (to undetectable levels). Other mostly downregulated genes were cytochrome b5-related protein, cytochrome b5-related protein-like, putative inorganic phosphate cotransporter, ecdysone oxidase-like and sorbitol dehydrogenase-like, whose expression decreased by between 45 and 173 times (Supplementary Table S2). Among the most upregulated genes during heat stress, we identified three isoforms of protein lethal (2) essential for life-like, whose expression increased by 7.4-, 2.4- and 7.7-fold compared to the control, as well as HSP70, whose expression increased 405-fold (Supplementary Table S2).

#### 2.2.3. Top 50 DEGs After No-Water Stress

Supplementary Table S3 shows the top DEGs were mainly enzymes and cuticle proteins. The most upregulated genes were fatty acyl-CoA reductase, glucose dehydrogenase (FAD, quinone)-like and larval cuticle protein A2B-like. The genes whose expression levels after the stress was not detected, thus considered 100% downregulation, were cholesterol 7-desaturase-like, arylphorin subunit alpha-like and fibroin heavy chain-like.

#### 2.2.4. Top 50 DEGs After No-Food Stress

After no-food stress, mainly enzymes, hormone receptors and vitellogenins were affected (Supplementary Table S4). One of the largest increases in expression level was noticed for cytochrome P450 6B6-like, with a level of 20-fold. The downregulated genes were two vitellogenin-like and probable nuclear hormone receptor HR3 isoforms X1 and X6.

### 2.3. Functional Analysis of DEGs

For GO classification, the DEGs of the four comparisons were divided into three categories: cellular component, molecular function and biological process (Figure 1C and Figure S1). Our GO enrichment analysis showed that the cold stress mostly enriched “cellular process and single-biological process” and “metabolic process” in the biological process, “binding and catalytic activity” in molecular function and “cell, cell part, membrane and membrane part” in the cellular component. The most enriched GO terms under heat stress were “metabolic process” in biological process, “binding” in molecular function and “membrane” in the cellular component. With the no-water stress, the most enriched pathways were the same as those under cold stress. In contrast, no-food stress mostly caused an enrichment of GO terms in “metabolic process” in biological process, “membrane” in the cellular component and “binding” in molecular function.

Under cold stress, the most enriched in metabolism were “fatty acid degradation” and “fatty acid metabolism”, while this was “drug metabolism-other enzymes” and “glycine, serine and threonine metabolism” under heat stress. Under no-water stress, the most enriched classification was the metabolism pathway at an organism level with “fatty acid degradation” and “drug metabolism-other enzymes”. In the no-food stress, this was “drug metabolism-other enzymes” and “glycine, serine and threonine metabolism”. Over the four stress conditions, KEGG analysis confirmed that cellular process, environment information processing, genetic information processing and metabolism were commonly enriched. Most DEGs belonged to fatty acid degradation, protein processing in the endoplasmic reticulum, ribosome biosynthesis in eukaryotes and protein processing in the endoplasmic reticulum under cold stress, no-water stress, heat stress and no-food stress, respectively (Supplementary Figure S2).

### 2.4. Identification of Genes Encoding for NPs, NPRs and Enzymes for BAs Synthesis

The conducted transcriptomic analysis allowed us to identify 56 genes encoding NP precursors, 58 genes encoding NPRs and six genes encoding enzymes responsible for biosynthesis of BAs (Table 3, Table 4 and Table 5). The expression patterns of identified genes varied depending on the stress type.

#### 2.4.1. Cold Stress

Out of all NP genes, the expression of six genes was upregulated during cold stress, while the rest (50 genes) were strongly inhibited. The gene most upregulated was *SIFa*, with an increase of 12-fold. This was followed by *Cal/DH*, *ITP*, *ITPlike*, *PDF* and *Trissin 2*—increases of 1.5–3-fold (Table 3). On the other hand, for *glycoprotein hormone alpha 2* and *ILP5*, there was a dramatic decrease with a total absence, representing a 100% decrease. This was followed by *AKH2*, *Allatostatin C* and *PBAN*, whose expression decreased by between 7 and 11 times (Table 3).

In the case of genes encoding NPRs, during cold stress, one-third of them (20) were upregulated, and 38 were downregulated. Among the overexpressed genes, the five most upregulated ones were *SIFaR*, *SPR*, *CalR1*, *PyrR* and *CAPA/PVKR*, whose expression increased by 2–7-fold (Table 4). ITPR expression increased from an undetectable level in the control to a low but detectable level during cold stress. The expression of other receptors such as *DH44R-1*, *CCHa1R-2*, *DH31/CalcitoninR-2*, *OrphanR-4*, *CCAPR-1*, *OrphanR-3*, *MSR-2*, *OrphanR-7*, *OrphanR-9, PABNR* and *NPFR* increased around one-fold (Table 4). Of the downregulated genes, the most profound effect was observable for *SKR*, which decreased by >22 times, and 17 genes were downregulated by between 2 and 7 times (Table 4).

We also analyzed the genes for crucial enzymes involved in BA biosynthesis, namely tyramine beta hydroxylase (*TβH*), histidine decarboxylase (*HDC*), tryptophan 5-hydroxylase (*TPH*), tyrosine decarboxylase (*TDC*), tyrosine hydroxylase (*TH*) and dopa-decarboxylase (*DDC*). During cold stress, *DDC*, *TH* and *HDC* were upregulated by 22-, 11- and 2-fold, respectively, while *TPH*, *TβH* and *TDC* were downregulated by between 3 and 5 times (Table 5).

#### 2.4.2. Heat Stress

Out of the NP genes, 22 were upregulated and 32 were downregulated under heat stress. The eight most upregulated genes were *CCAP*, *PDF*, *Trissin-2*, *SIFa*, *NPF 2*, *RYa*, *CCHa2* and *NPF 1b* with an increase of about 2–3-fold (Table 3). *NPF*, *ASTA* and *ILP4* showed an increase of about 30%. In the case of *ETH*, its expression diminished to undetectable levels during heat stress (total downregulation), and *EH* was downregulated by about 5 times. The second most downregulated NP was *GPH alpha 2*, which diminished by 9 times, and this was 2–3 times lower for *ILP5* and *AST CC*.

In the case of the genes encoding NPRs, during heat stress, 18 were upregulated and 40 were downregulated. Among the overexpressed genes, *ITPR* and *Orphan R-10* were not detected in the control, while their expression was notable in stressed insects. *CAPA-PVKR1 ILPR-1*, *SPR* and *SKR* were overexpressed with an increase of 2–4-fold. On the other hand, the expression of *ACPR-2* and *CCHa1R-2* diminished to undetectable levels during heat stress. The next most downregulated receptors were *AKHR*, *DH31/Calcitonin R-1*, *CAPA-PVKR-2*, *DH44R-2*, *DH44R-1*, *AST-C R* and *RYaR-1*, whose expression decreased by 2 to 5 times (Table 4).

We observed an upregulation of genes associated with the synthesis of histamine—HDC—by 3-fold. On the other hand, the genes associated with the synthesis of OA and DA were downregulated, *TβH* and *TH* both, by about 6-fold (Table 5).

#### 2.4.3. No-Water Stress

Among the NP genes, in 15 genes, the expression increased, whereas in 40 genes, there was a decrease. Two of the most upregulated genes were *OrcA* and *CCHa2*—by 2-fold (Table 3). Also, *ITP* and *ITPL* were upregulated by almost 2 times. *AKH* and *NPF1b* were also upregulated, however, with a lower efficacy (<1-fold). On the other hand, the most downregulated were genes for *ETH*, *GPB5*, *ILP5* and *SK*, which decreased by between 8 and 3 times. A group of genes, including *CCH1a*, *NPs*, *ILP6* and *DH*, were downregulated by about 2 times. For *GPB5* and *SIFa*, the expression was below the detection limit, and thus the inhibition of expression was the total.

For the NPRs, 26 genes were found to be upregulated, whereas 28 were downregulated. For *SKR*, *ACPR* and *CCHa1R*, no changes were detected. The most upregulated NPRs were genes for *Cal/DHR1* and *SIFR*—more than 2-fold. The genes for *NPFR*, *SPR* and *ACPR-2* were upregulated by 1-fold. The expression levels for *ITPR* and *CAPA/PVKR* were not detected after no-water stress. The most decreased expression level was noted for *AKHR*—5-fold. A group of NPRs with *natalisin receptor*, *CCAPR*, *PDFR*, *CCHa1R*, *RYaR-1*, *RYaR-2*, *PTTHR*, *EHR*, *CNMR* and *sNPFR-2* were downregulated by around 2–3-fold. The expression level of other NPR genes was dropped down but with a lower degree (Table 4).

In the BA synthesis genes, *TDC* and *TβH* were downregulated. A stronger downregulation (5-fold) was observed for *TβH*, whereas *TDC* expression dropped down >1-fold. Genes encoding enzymes involved in DA synthesis were, in contrast to OA synthesis, upregulated. *TH* was upregulated by 5-fold and *DDC* by almost 14-fold. Different results were noticed when the genes encoding enzymes involved in serotonin biosynthesis were analyzed. The expression level of *TPH* decreased by 2 times, while *DDC* expression increased 14-fold. The gene encoding *HDC* was upregulated, similarly to OA-related genes, but only by 2 times (Table 5).

#### 2.4.4. No-Food Stress

The expression level of 48 NP genes increased, whereas a decrease was observed for only five genes. The expression levels of *GPB5*, *ILP5* and *Cal/DH* did not change after starvation (Table 3). The most upregulated NP genes were genes for *SIFa* (increased almost 10-fold), *CCAP* (increased more than 4-fold), *PDF*, *Triss2* and *RYa* (2-fold increase). The group of NP genes with a 1-fold increase included genes for *AKH2*, *ACP*, *NPF1b* and a few others. Further, some genes were upregulated by <1-fold (Table 3). Among the NP genes that are downregulated, the largest decrease, by almost 2 times, was observed for *ETH*. Other downregulated NP genes were *ILP3*, *BurB*, *GPBA2* and *SK*.

For the NPRs, 26 genes were upregulated, whereas 28 were downregulated (Table 4). The most upregulated NPR gene was the one for *ACPR*. The second most strongly upregulated was the gene for orphan *Receptor 4*, classified as an NPR based on sequence similarity—>2-fold (Table 4). Two genes, *CH/DHR* and *TRPR*, were upregulated 1-fold, whereas the others were upregulated <1-fold. For the *CAPA/PVKR*, *NTLR*, *PDFR* and *CCHa2R* genes, no changes in expression were detected.

In the genes involved in BA synthesis, a significant upregulation was detected for *DDC*, about a 1-fold increase. The expression level of genes from the 5HT biosynthesis pathway encoding *TPH* and *DDC* also increased. The *DDC* gene expression increased more strongly, almost 1-fold, but the *TPH* gene expression was only 0.3-fold. An increase was also observed for *HDC*, which was upregulated >7-fold. The only downregulated gene was for *TβH*, which is involved in OA synthesis (a 2-fold decrease); however, the second OA pathway gene encoding TDC was surprisingly slightly upregulated (Table 5).

### 2.5. Multivariate Analysis

**Figure 2:**
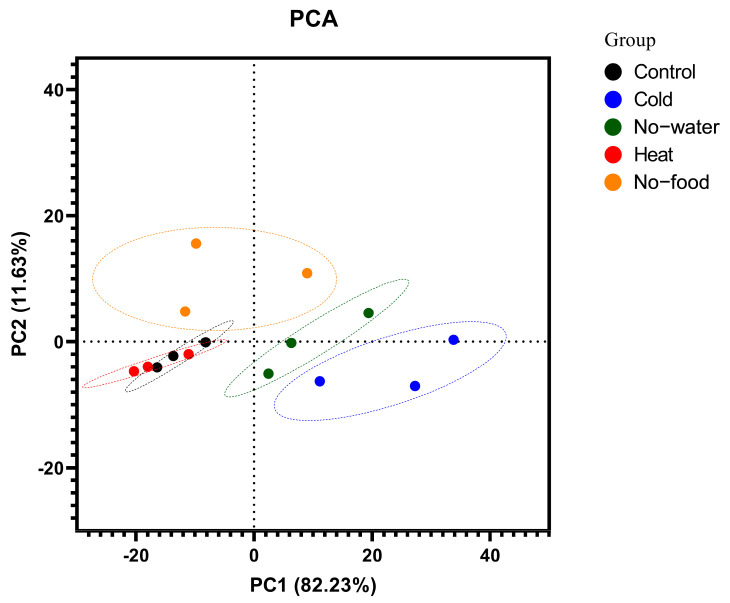
Principal component analysis (PCA) displaying the two first principal components (PC1 vs. PC2) based on expression profiles of neuropeptides (NPs) identified in the brain of *Spodoptera frugiperda* after Cold, Heat, No-water and No-food stresses. The first two principal components (PCs) comprise 93.86% of the variability—82.23% and 11.63% for PCs 1 and 2, respectively.

Using the data on the expression of identified NPs, we conducted a principal component analysis (PCA) to analyze brain-specific genotypes among all experimental variants. The cold, no-food and no-water treatments were clearly separated along the first principal component (PC1). The first two PCs accounted for 93.86% of variance with PC1 explaining 82.23% and PC2 11.63%. The cold and no-water stress showed similar patterns of expression, yet separated from each other, while the control and heat stress grouped together. The most divergent from all others was no-food stress, which showed separation along PC2 between cold, heat and no-water stresses (Figure 2). Most of the genes correlated negatively to PC1, and the ones that correlated most negatively to PC1 were genes encoding neuroparsin, sulfakinin, ILP4 and ASTB. Only five genes correlated positively to PC1, namely *ITP-like*, *ITP*, *EH*, *SIFa* and *DH30/Cal*. Only *ETH*, *DH30/Cal* and *Glycoprotein hormone ꞵ* correlated negatively to PC2, while all the others correlated positively, with *Trissin-1*, *IMFa* and *allatotropin-like peptide* being most positively correlated to PC2 (Figure S3).

## 3. Discussion

In nature, insects are exposed to many intertwining stress factors affecting many physiological parameters. Key stresses that insects have to overcome are frequent temperature changes as well as water and food availability. The ability to adapt to different types of stresses has made *S. frugiperda* able to inhabit a wide range of environments. In this study, we conducted a comprehensive transcriptome and neuroendocrinome analysis and characterized the gene expression profiles of *S. frugiperda* under four different stress types: cold, heat, no water and no food. Also, given today’s global climate change and the significant loss in insect biodiversity [22], we aim to better understand the impact of thermal, no-food and dry stress on insect development, population dynamics and feeding behavior.

### 3.1. Responses to Temperature Changes with Cold and Heat Stress

Temperature stress is undoubtedly one of the most important abiotic stressors influencing insect physiology. Insects are poikilothermic, and in turn, their body heat is derived exclusively from their external environment. In general, insects can live under a wide range of thermally stressed climates, for instance, from Antarctica (capable of surviving even −196 °C) to temperatures near boiling; however, the optimal temperature for breeding and growth is in the range of 25 to 32 °C [23]. As a consequence, an important aspect of the response to thermal stress is the modulation of major metabolic pathways, such as glycolysis, the TCA cycle or β-oxidation, which regulate energy metabolism [7]. These adjustments in metabolic processes are necessary to evade and prevent the detrimental effects of cold stress. Hence, the synthesis of fatty acids and cryoprotectants (amino acids and sugars) can be observed [24]. The changes in lipid levels and composition seem extremely important to maintain cell membrane fluidity, which is a first line of defense against cold stress and energy production [24,25]. Our results confirmed the above hypothesis, as the most enriched genes in *S. frugiperda* during cold were the “metabolic process” in biological processes and the “cell, cell part, membrane and membrane part” in the cellular components. In turn, in metabolic processes, the fatty acid metabolism and degradation were most enriched, as well as those in the top DEGs, such as putative fatty acyl-CoA reductase. As the stress–response pathways are interactive and cross-talk, immune-related mechanisms are also efficient in cold stress tolerance [26]. Among the top DEGs, we found the upregulation of *mucin-5AC*. Although mucins are highly glycosylated macromolecule proteins secreted by various epithelial cells and are distributed on the surface of the lumen in the body [27], they have recently been proven to take part in immune response [28]. When experiencing critical cold stress, insects enter a comatose state known as chill coma, which is characterized by complete neuromuscular paralysis. This state is reversible when the temperature rises back to an optimal level [29]. The chill coma is primarily caused by imbalances in ion homeostasis, specifically hyperkalemia (increased K^+^) [4]. The observed downregulation of most *NPs* in *S. frugiperda* under cold stress is associated with the chill coma, as the entrance into this state is linked with neuronal silencing of the metathoracic ganglion and brain in insects, as has been seen in locusts and flies [30,31]. This silencing of the nervous system during cold stress is associated with cell depolarization due to a sudden increase in extracellular K^+^ surrounding the neurons. The mechanisms that are responsible for controlling ion homeostasis are complex in insects. This includes the regulation of the activity of Malpighian tubules and the gut, as well as the volume of the hemolymph [32]. However, what their effect is on the growth, development and behavior of insects, eventually under thermal temperature stress, remains unknown.

**Figure 3:**
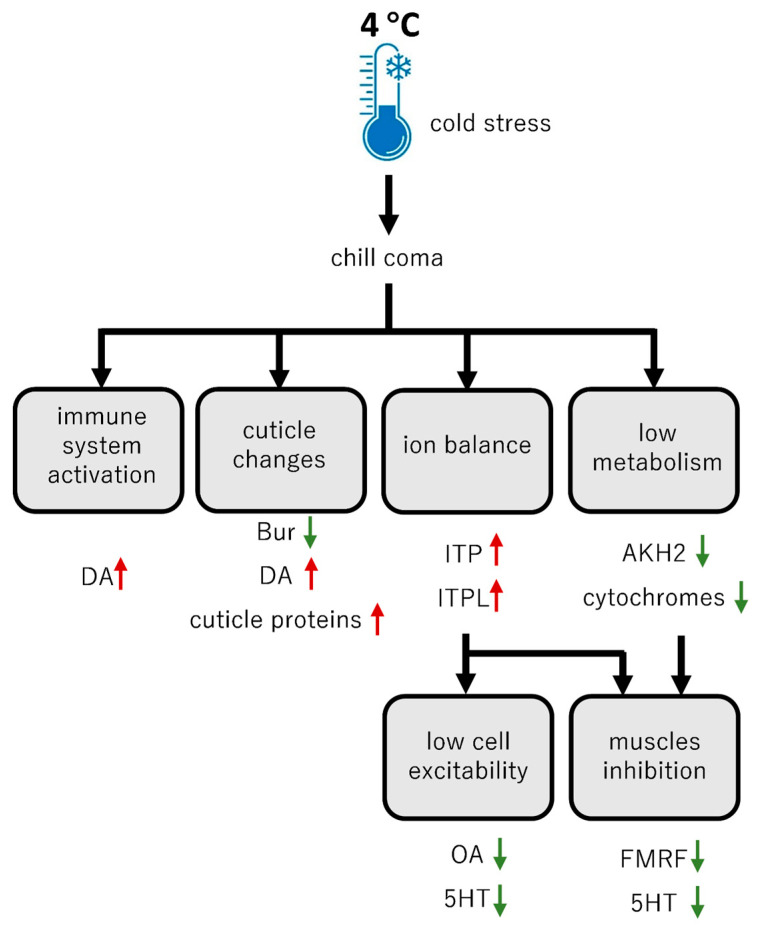
Cartoon model with involvement of brain neuropeptides (NPs) and biogenic amines (BAs) in the regulation of *S. frugiperda* physiological processes in cold stress. The green arrows indicate downregulation while the red arrows upregulation.

On the ion homeostasis control, the ITP and ITP-like are involved in Cl^−^, Na^+^ and Ca^2+^ transport and fluid reabsorption [33], while the Cal/DH stimulates diuresis in the Malpighian tubules [34] (Figure 3). As the chill coma is characterized by a complete neuromuscular failure, the maintenance of motoneuron activity would be crucial for chill coma recovery. Also, Trissin-2 takes part in foregut and midgut contractions, affecting water balance, and it is co-expressed with Cal/DH and CRF/DH in the brain of *B. mori* [35]. Most of the upregulated genes also take part in the circadian clock regulation, which monitors daily and seasonal light and temperature cycles and which entrains behavioral and physiological rhythms to match with them, and it could also play a vital role in cold tolerance [36]. Intriguingly, in *Drosophila montana* flies, the cold tolerance traits are under photoperiodic regulation [37]. When bringing circadian organisms such as *Drosophila* under extremely high- or low-temperature conditions, the rhythmic cycles are abolished, which may explain the increase in the level of NPs responsible for maintaining the circadian clock to return to normal rhythmicity after cold stress. This notion can be supported by the abovementioned fact that *PDF* was overexpressed in the brain of *S. frugiperda.* PDF-expressing lateral neurons (s-LNv) are the master pacemaker neurons in the circadian hierarchy [38,39]. The PDF signaling from the s-LN_v_ neurons controls two PDF receptor (PDF-R)-positive LN_d_ neurons and sets their free-running periods [40,41]. Taking this into account that our data with *S. frugiperda* showed an increase in the expression of *NPs* such as *Cal/DH*, *ITP* and *ITP-like*, which regulate ion balance and their receptors, and *PDF* as a response to cold and heat stress, we think this may be a counter-measure to act against the detrimental effects of thermal stress. On the other hand, we observed a decrease in *FMRFa* expression, which might be important for the recovery from chill coma. The latter hypothesis is supported by the fact that in *Caenorhabditis elegans*, the release of *FMRFa-like* NPs, encoded by the *flp-13* gene, is required for anterior lateral neuron activation during cold and heat stresses, which promote quiescence; hence, it has to be suppressed for the recovery period [42]. Our study confirms the role of *Cal*/*DH, ITP* and *ITP-like* in thermal stress. Further studies, also in other insects, should investigate the mechanisms involved.

In BAs, we found that during cold stress, the expression of *TDC* and *TβH* decreases, suggesting that levels of OA and TA drop down in the insect body. Both of these amines are involved in the regulation of different physiological processes from fat metabolism, circadian rhythm, fight or flight response and muscle regulation. Indirectly, OA regulates the ion flows. The activation of the OA receptor is followed by a cascade of secondary signals leading to increased activity of cAMP-dependent protein kinase A, which in turn regulates the activity of K^+^ channels and Na^+^/K^+^-ATPase. Hence, the lowered cell excitability during chill coma may be partially caused by lowered levels of OA [43] (Figure 2). Another amine, whose levels seem to be changing, is DA. Based on the obtained results of the increased expression of *TH* and *DDC*, the levels of DA are elevated during cold stress. Firstly, DA takes part in cuticle melanization [44,45]. Melanization is an adaptation to cold stress, and the darker-body phenotype associated with this process increases cold hardiness by allowing the animals to absorb more heat from solar radiation (thermal melanin hypothesis) [46]. Secondly, melanin is also an important part of the insect immune system, as the product of TH-L-DOPA is a substrate for phenoloxidase (i.e., one of the key enzymes of the innate immune system in insects) [26,47]. The DA might also regulate another part of the insect immunity, namely the cellular response by affecting phagocytosis through the AC-cAMP-PKA–NF–κB signaling pathway, as shown by Tong et al. [48].

On the other end of the thermal stress axis is heat stress. The nervous system plays a fundamental role in perceiving high temperatures and appropriately responding to them by changing the physiology and behavior of an animal. Heat stress response starts with temperature sensing via thermoreceptor neurons from the peripheral nervous system. Thermoreceptor neurons respond to action potentials driven by changes in the concentrations of ions (e.g., Na^+^, K^+^, Ca^2+^ and Mg^2+^) across the membrane [9], while the heat stupor is accompanied by drastic increases of extracellular K^+^ concentration and electrical silence in the CNS (similar to chill coma) [31]. However, in our experiments, the caterpillars of *S. frugiperda* did not enter a heat coma at the high temperature of 42 °C, and there was also no loss of survival. The observed decrease in the expression of *DHs* is most likely the mechanism responsible for this. DHs increase the excretion of water through the Malpighian tubules [49,50]. The decrease in their expression is aimed at retaining water in the body and thus probably reducing the K^+^ concentration to maintain proper neuronal functioning. Also, similarly to chill coma, FMRFa and Orcockinin promote quiescence during heat stress. OrcA shows more profound effects than FMRFa in *C. elegans* [51], while in *T. castaneum*, *OrcA* is considered an awakening factor [52]. On the other hand, *Drosophila* responds to heat stress with an increase in sleep. A sleep-regulating role for a signaling pathway involving FMRFa neuropeptides and FMRFa receptor (FMRFR) has been demonstrated [53]. The authors demonstrated that flies with silenced *FMRFa* or *FMRFR* showed a shorter sleeping time in response to high temperature stress. Hence, it has to be suppressed for the recovery from the coma. In our experiments, the genes encoding both of these peptides are suppressed in the brain of *S. frugiperda*, indicating that this is a mechanism to prevent entering into a heat stupor; however, further study is required to clarify the mechanism behind it.

Since the resting metabolic rate (RMR) increases significantly with higher temperatures [5,54], regions with high baseline temperatures are particularly dangerous under changing climate conditions even if the increase in temperature is low. Therefore, reducing the metabolic rate is an important strategy to escape stressful conditions, such as extreme heat [55]. In our experiments, we observed a downregulation of genes such as *ILPs*, *AKH*, *SK* and *ASTCC*, indicating that this happens also in *S. frugiperda*. During heat waves, insects are in a state of high energy demand to properly function and withstand the stress. As a response, the anaerobic metabolism can be recruited as a complementary source of energy that can increase insect survival at temperatures close to survival limits [56]. As this type of oxidation is less efficient at producing a smaller amount of energy [57], an increase in food consumption should be accompanied. In our experiments with *S. frugiperda*, an overexpression of NPs responsible for food intake was observed, exemplified with *sNPF* [58], *NPF2* [59] and *SIFa* [60], supporting this notion (Figure 2). On the other hand, we also observed an overexpression of *CCHa-2* after heat stress. CCHamide has first been reported as a brain–gut NP, which is specific in insects and associated with the regulation of feeding behavior [61]. Recently, it has been shown that CCHa2 signaling inhibits feeding in *Gryllus bimaculatus* [62], while in *D. melanogaster*, this isoform increases feeding motivation [63]. Additionally, the second isoform of CCHa, namely CCHa-1, is responsible for the regulation of olfaction [64] and circadian activity [65]. This shows that governing feeding behavior in insects is complex and further analyses are needed to elucidate the role of these peptides during heat stress.

During heat stress in insects, the nervous system is protected by an increased expression of heat shock proteins (HSPs), especially in the perineurium, glia and neural membranes [55]. These proteins are molecular chaperones that prevent the denaturation of other proteins that cannot remain active at high temperatures. As expected, also in *S. frugiperda*, there is an increased expression of genes encoding proteins belonging to the HSP family, such as *protein lethal (2) essential for life-like*, which is a member of the HSP20 family, and HSP70, the most commonly described in the insect heat response [55].

The genes that are strongly inhibited during heat stress are the ones that are responsible for ecdysis and eclosion in the molting process of the insect, namely *EH*, *ETH, CCAP* and *E20MO* [6,66]. In addition, we saw a simultaneous increase in the expression of genes responsible for the inactivation of ecdysteroid molting hormone, namely *E20O* [67,68]. A delay in ecdysone secretion and an increase in JH content, resulting in delayed metamorphosis or additional molting, is a common phenomenon in insects subjected to high temperatures, which allows the stressed individuals to “wait out” the unfavorable conditions [69]. Interestingly, these changes are also accompanied by an increase in DA and OA with a simultaneous decrease in the activity of their metabolic enzymes [69]. Our data in *S. frugiperda* confirmed these observations. Apart from genes from histamine pathways, all other genes involved in DA and OA biosynthesis were downregulated (*TβH*, *TDC* and *TH*). Nonetheless, further research should focus on determining the level of these BAs during heat stress and correlate them with the enzymatic activity of TH and TDC.

### 3.2. Responses to No-Water Stress

One of the key environmental stresses that insects face in both natural and agriculture and forestry ecosystems can be dehydration [70]. Maintaining water homeostasis is crucial for proper functioning of the body. Thus, insects have evolved several physiological and behavioral adaptations to conserve water [71]. This includes a water-impermeable cuticle that is covered with a thin layer of epicuticular lipids, which reduce cuticular permeability [71]. To orchestrate all processes and particularly adapt to periods of drought or limited access to water, a properly functioning brain, sending signals to other tissues, is crucial. On the other hand, dehydration can cause dysfunction of the insect brain. At the molecular level, if water levels are too low, the water and ion homeostasis balance can be disturbed, and the brain cells work harder to complete tasks. This is shown in *S. frugiperda* transcriptome analysis where the DEG numbers are significantly higher during water deprivation stress than with the other stresses of temperature and no food. Moreover, the functional analysis showed that these are mainly genes involved in metabolic pathways, such as fatty acid degradation, drug metabolism and other enzymes. This may also indicate an increase in metabolic water production. Indeed, this assumption is confirmed, as the most upregulated gene in our research was fatty acyl-CoA reductase. Further studies are required to clarify the mechanism.

Deregulation in water homeostasis could also be seen when we analyzed the neuroendocrinome of *S. frugiperda*. Two of the upregulated genes were *ITP* and *ITP-like.* In insects, these NPs have been shown to be involved in Cl^−^, Na^+^ and Ca^2+^ transport, as well as fluid reabsorption function [33]. Moreover, in *D. melanogaster*, ITP was proposed as a master regulator of water homeostasis, where its levels increase under no-water stress, thus protecting the flies from water loss by increasing thirst, reducing the excretion rate and promoting ingestion of water instead of food [72]. This function was also confirmed in *B. mori* [33]. In our experiment, the elevated expression of *ITP* clearly confirmed that the insects suffer from water stress.

The group of upregulated genes after limited access to water contained NPs that are involved in the regulation of food intake and metabolism, e.g., *CCHa2*, *AKH* and *NPF1b*. This again indicated that the signals from the brain might be responsible for metabolic response, which relays the activation of reserves to obtain metabolic water and/or use the obtained energy to escape from unfavorable conditions, especially in short, acute stress, as was in our experiment. The highly upregulated expression of *OrcA* might also confirm this hypothesis, as orcokinins are known in various species to be involved in circadian cycle functioning, and in *T. castaneum*, it has been proposed as a factor inducing “awakening” activity [49]. Furthermore, the locomotory rhythms are also controlled by ITP in *D. melanogaster* [73]. Taken together, we believe that our data showed that the no-water stress promoted the insect to increase the reserve mobilization to gain metabolic water and search for moisture in the environment. However, further studies are required to clarify the mechanism, and the involvement of a circadian cycle functioning can be very intriguing.

Changes in genes related to the BA biosynthesis pathways were also observed after no-water stress. In general, in insects, the BA levels increase during various stresses, correlating with a downregulation in the expression of these genes involved in their biosynthesis [69]. In our experiments, it was noted that genes involved in DA biosynthesis were downregulated. This is correlated with the elevated expression of *NPF2*, as it was shown that this signaling pathway interacts with the DA pathway [74]. DA can be involved in cuticle melanization, and in turn, melanin is engaged not only in cuticle pigmentation but it also affects the cuticular permeability [75]. Thus, elevated genes of the DA synthesis pathway can be another organismal response to limited water loss conditions. Surprisingly, the OA level probably is not elevated after no-water stress. The reason for this is unclear and needs further elucidation.

### 3.3. Responses to No-Food Stress

Starvation can be described as the physiological state of an organism with a low or stopped energy intake. As a consequence, starvation can impair the proper functioning of crucial organs in the insect body, such as the brain. The brain of all animals is the main regulatory center combining nervous and endocrine signaling, thus adjusting all physiological processes. In addition, the brain tissue itself is one of the most energy-demanding tissues in the organism [76]. Based on these arguments, one can suppose that an inadequate energy supply, as under no-food stress, will have tremendous effects on the proper brain functioning and will trigger signaling changes that will affect metabolic processes in other parts of the insect body. Our results agree with this hypothesis, showing notable changes in the profile of gene expression after no-food stress. The most prominent changes were observed in genes associated with metabolic pathways at an organismal level, such as drug metabolism and other enzymes or glycine, serine and threonine metabolism. Similar research was recently performed in another lepidopteran insect, *B. mori*, where it was also shown that after a period of starvation, the most prominent changes in gene expression in the brain were associated with nutrition metabolism and energy metabolism [77]. This is also confirmed in our experiment with the list of the top 50 of up- and downregulated DEGs after no-food stress, where significant changes were shown in genes for cellular metabolism, such as genes encoding cytochrome P450, dehydrogenase and acyl-CoA reductase.

Among genes for metabolic NPs after no-food stress, we found an upregulation of *SIFa*, *CCAP*, *PDF*, *Triss2*, *RYa*, *CCHa2*, *AKH2* and *NPF1b*. As shown in *D. melanogaster*, the major neuroendocrine stress response embraces, apart from BAs, NPs such as AKH and ILP [60]. AKH, which was shown to be upregulated in our research after no-food stress, regulates metabolic responses to various stresses [78]. It was shown to stimulate catabolic reactions and mobilize energy stores, especially lipids (triglycerides) and trehalose [69,78]. The AKH-producing cells (ACP) in the nervous system of the insects were shown to be nutrient sensitive and release AKH in low-energy conditions and high-energy-demand conditions [69]. A significant upregulation of the *AKH* gene after no-food stress can undoubtedly be an indicator that the insect is trying to increase its metabolic rate in order to survive unfavorable conditions. On the other hand, AKH in the brain was proven to modulate insect behavior—mainly increasing the locomotory activity and gustatory sensitivity in order to search for a novel source of energy. The other NPs, commonly known to be involved in the regulation of feeding and metabolism, are NPF and others [59]. These were shown to increase the lower quality food intake, and the food might be less favorable or even be noxious when the insect is experiencing a hard period of starvation [59]. We note here that *S. frugiperda* has a polyphagous eating behavior. Moreover, the NPF signaling system was shown to be crucial for proper odorant detection and sensitization of olfactory neurons. This can be crucial during a no-food stress period in order to facilitate food seeking. Another NP involved in the regulation of food-seeking behavior based on metabolic state is SIFa. It was shown that neurons expressing SIFa in the brain of *Drosophila* induce food uptake, receiving signals from other brain cells [60]. Locomotory activity and circadian clock are also regulated by another neuropeptide PDF, which is highly upregulated [79]. Thus, the neuroendocrinome response in the brain of *S. frugiperda* may have led to an increase in metabolic rate and locomotor activity in order to escape from unfavorable conditions or quickly search for a new food source.

The second group of neuromolecules in the brain involved in response to stress conditions are BAs, mainly OA and DA [69]. After no-food stress, the DA pathway was partially upregulated. It was shown that dopaminergic neurons contain NPFR in the nervous system of *D. melanogaster* larvae [80], suggesting that NPF and DA signaling pathways interplay in the regulation of various processes, such as appetitive memory and olfaction. Moreover, DA modulated the starvation-induced sugar response after a short starvation period [81]. OA and TA, the other biogenic amines, have been reported to promote feeding behavior [82]. Starvation, which is considered a stressor, should also trigger an increase in amine levels, and our data confirmed this. The expression of *TBH* decreased after no-food stress, while that of *TDC* responsible for TA biosynthesis increased. Probably, after 12 h, the OA pathway was activated. We expect that a longer starvation duration might lead to an upregulated *TBH* expression in the brain [83].

### 3.4. The Insect Brain Responding to Stress Conditions

The brain in insects is the major organ where neurohormones such as NPs and BAs are produced. They can work locally as neurotransmitters or as classical hormones, regulating a vast array of physiological processes, including the growth and development of the insect [84]. Thus, we analyzed the neuroendocrinome to check whether we can observe changes in the expression of NP genes involved in food and metabolism, locomotory activity and growth and development. Using the data gathered on the expression of NPs, we conducted a PCA analysis to identify if responses to abovementioned four stresses share common expression patterns. PCA is the most prominent dimension reduction approach that can be used to ease regression analysis, where just a limited number of PCs, instead of the original measures, are utilized as variables [85], and it has been widely used in the analysis of gene expression [86]. There was no separation between control insects and heat-stressed, control and no-food insects. Despite the fact that cold stress is often accompanied by desiccation [2], the analysis showed a separation of brain responses to cold and no-water stresses. Similarly, when comparing no water with no food, a clear-cut separation could be observed. From the transcriptome data of *S. frugiperda*, it can be seen that the most different response occurs in cold stress. Also, our analysis allowed us to conclude that the responses to cold exposure and food deprivation differ the most. Considering the differences between all stresses, it emerges from the PCA analysis that in order to distinguish between different types of stresses, future studies may focus on the expression/level of *ITG*, *LQDVa*, *ETH*, *PBAN*, *ILP*, *DHs*, *ITP* and *ITP*-*like*, as they contribute the most to the PCA separation structure (Figure S3). In particular, the focus should be on the comparison of *EH* vs. *ITP* and *ITP*-*like*, as they are inversely (negatively) correlated and are positioned on opposite sides of plot origin in diagonally opposed quadrants (Figure S3). These genes then may be considered good candidates as markers to distinguish particular types of stresses (i.e., cold vs. heat), while the *ITG* vs. *ITP* and *ITP*-*like* could be good markers to differentiate no-food and cold in *S. frugiperda*. Taken together, *ITP* and *ITP*-*like* can be used as markers for cold and water stresses, *ITG* and *LQDVa* can be used for no-food and *EH* can be used for heat stress.

### 3.5. Effect of Stress on Insect Feeding, Metabolism, Size and Shelter Behavior

What happens with the insect during thermal stress and when stressed by drought and starvation, and how can the insect adapt and escape from these stresses? To provide insight into the different physiological status that insects occupy during cold exposure, we can compare it with studies using diapausing species, which are species that are prepared for cold exposure and that enter a hypometabolic state [5,87]. Controlling the metabolic rate is fundamental to avoiding extreme temperature stress conditions. The important mechanism for regulating metabolic rate is protein phosphorylation by regulating transcriptional and translational factors to control fuel metabolism [88]. Additionally, our experiments have shown an activation of the immune system during cold stress, as evidenced by mucin. However, it remains unclear why this occurs and whether it is a response to damage. All in all, we can conclude here that metabolic rates usually decrease with a decrease in temperature and an increase in body size. In cold climates, insects grow to larger body sizes compared to hot climates [89]. These data increase our basic understanding of the “cross-talk” between different metabolism-related systems and cold stress, but further studies should investigate the mechanism(s) involved.

For heat stress, changes in metabolic behavior in poikilotherm insects can also be considered a delayed response. It has been shown that significant changes in feeding intensity as well as in lipid content in *D. melanogaster* females occur 24 h after short-term heat exposure (38 °C, 60 min), resulting in a decrease in food consumption [90,91,92]. These data suggest that the regulation of feeding behavior under stress may be controlled by mechanisms other than the IIS pathway. Indeed, Ugrankar et al. [93] observed that there are changes in the expression of the pathway’s genes, and the increase in the carbohydrate level takes place directly after short-term heat exposure. Also, the behavioral response manifests as a decrease in food consumption, resulting in a decrease in lipid content. Our findings in the invasive *S. frugiperda* showed the effect of the insulin pathway elements with ILP, ILP-like and InR. However, after heat stress, we only observed the upregulation of *ILPR1*, whereas there were no significant changes in *ILPs*. After starvation, the upregulation of *ILPR2* and *ILPR3* was observed to be accompanied by an increase in *ILP1*. However, what their effect is on the physiology with the body size and behavior of insects, including under heat stress, remains unknown. Also, high thermal stress may affect the insect’s reproduction and fecundity. It was also seen in our experiments that genes encoding hormones involved in the regulation of molting and reproduction, such as *ETH*, *EH*, *ASTCC* and *ASTA*, were downregulated. We believe this hypothesis deserves to be tested with other insects, including beneficial insects such as pollinators and natural enemies, and also in other stress conditions like pathogen infection.

Regarding the metabolic mechanism, insects consume energy to support their survival [88]. But long intervals of high metabolic rates result in the death of the insect because of energy depletion followed by great oxygen consumption, which causes oxidative damage to lipids, proteins and nucleic acids [5,87,94]. To avoid cellular damage in temperature stress, insects can use antioxidants such as glutathione-S-transferase, superoxide dismutase, ascorbic acid and thiols in association with physiological stress responses. Controlling the metabolic rate is fundamental to avoiding extreme temperature stress conditions. The important mechanism for regulating metabolic rate is protein phosphorylation by regulating transcriptional and translational factors to control fuel metabolism. Metabolic rates usually increase with an increase in temperature and body size. In hot climates, insects grow in smaller body sizes compared to cold climates. For instance, beetles grew with smaller body sizes by 1–3% with every rise of 1 °C [5]. Another example of body size changes in our current agroecosystem is that, at the community level, species with a stable or increasing relative abundance tend to be larger than declining species [95]. We believe that future studies on metabolic mechanisms are useful for documenting the potential markers of global change effects.

Shelter-building behavior in insects is another important adaptation [5]. The shelter-building behavior of insects enables them to resist extreme temperatures and environmental changes. Due to shelters, the insects would be able to survive their desiccation period in the dry seasonal spells. Indeed, silk secretion is a characteristic behavior of 98% of Lepidopterans, and larvae use silk strands to draw leaf surfaces together to make a shelter [96]. This behavior can go together with the fact that insects develop in different seasons and so are faced with different food availability and suitability, as well as environmental conditions [5]. Moreover, the pattern of the insects to use the host may be linked with voltinism because some host plants may be the better choice and can help in the production of additional generations. Such behavior has been seen with different insects, including *S. frugiperda* [97,98]. Increased food consumption has been observed with the increase in temperature, which resulted in higher rates of development [5,99]. A more rapid life cycle and a reduction in total developmental time can lead to a reduction in exposure to natural enemies, resulting in increased infestation and damage. We think this hypothesis should also be tested with other insects.

It is clear that food and temperature are the most important factors among those that affect the overall distribution of plants and wild animals, including insects [5,100]. Battisti et al. [101] and Bodlah et al. [5] reported that under rapid climatic anomalies, insect species expand their ranges, and as a consequence, non-host plant species can face sudden damage. The insect of our study here, *S. frugiperda*, is a polyphagous invasive Lepidopteran. We believe that future experiments on host preferences and dispersal, including under different environmental conditions, may help to better understand the range expansions that this invasive pest has done in the different countries in the Americas, Africa and Europe. Today, however, we have no information for *S. frugiperda* in terms of whether an increase in temperature may increase the larval survival in different host plants and, thus, its dispersal—for instance, to enter Europe. Interestingly, a previous study by Braschler and Hill [102] demonstrated that insect species may have a great chance to track climate changes by incorporating or switching to other and novel host plants. Future molecular ecology studies are required to investigate this.

**Figure 4:**
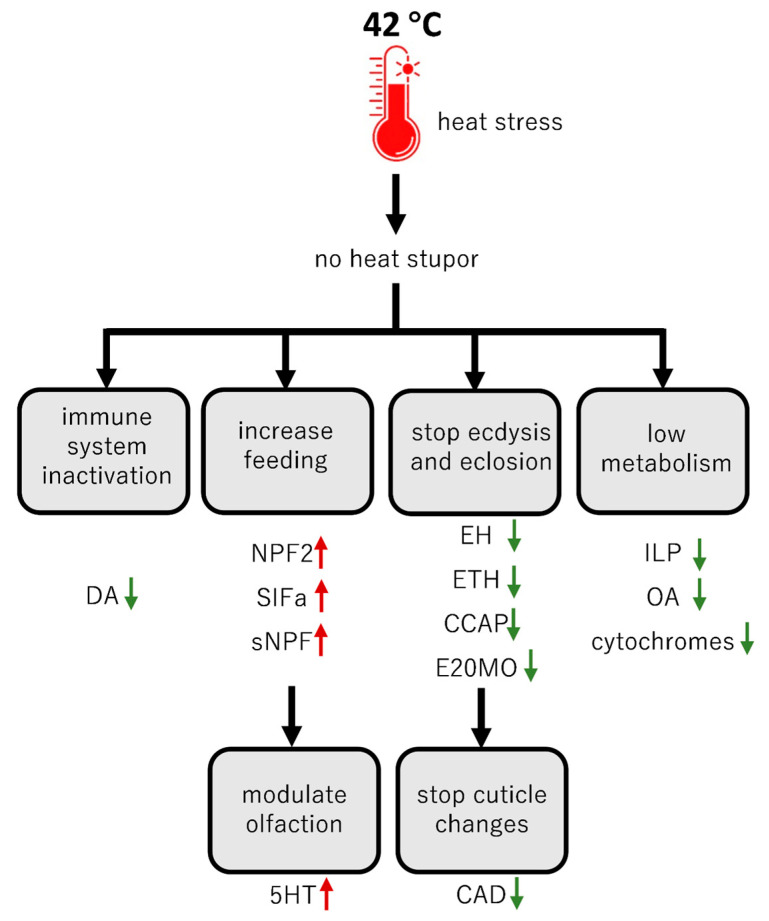
Model with involvement of brain neuropeptides (NPs) and biogenic amines (BAs) in the regulation of *S. frugiperda* physiological processes in heat stress. The green arrows indicate downregulation while the red arrows upregulation.

Finally, our data may also increase our basic understanding of a potential hormesis effect [103]. Hormesis is a two-phased dose–response relationship to an environmental agent whereby low-dose amounts have a beneficial effect and high-dose amounts are either inhibitory to functioning or toxic. For instance, beneficial effects can include an increased life span, improved rates of growth and development and increased resistance to infection and tolerance to pesticides [104,105]. In our experiments with *S. frugiperda*, we can speculate about the activation of the immune system during cold stress or the increase of feeding in heat stress (Figure 3 and Figure 4). We believe this hypothesis deserves to be tested with other insects and also in other stress conditions, such as pathogen infection and pesticide exposure.

## 4. Materials and Methods

### 4.1. Insects, Experimental Setup and RNA Sequencing

The larvae of *S. frugiperda* were collected in corn fields at Guizhou Academy of Agricultural Sciences on 20 June 2019. The insects were reared at 25 ± 1 °C and 75 ± 5% RH in a photo period with 16 h light and 8 h darkness [106].

For the experiments, we kept 4th instar larvae under five different conditions: (i) normal conditions as above (Control), (ii) cold stress of 4 °C for 2 h (Cold), (iii) heat stress of 42 °C for 2 h (Heat), (iv) no-water stress for 12 h due to limited access to water where no food and no water were provided (No-water), and (v) no-food stress for 12 h where no food was provided but water was provided with wet cotton (No-food). The cold stress procedure induced a chill coma in the insects with 100% recovery from coma upon the return to control conditions, and no treatments caused mortality among the insects. We cut off the head, including the brain, of these larvae and prepared them for RNA sequencing. The experiment was performed with three biological replicates, and each biological replicate consisted of five heads (brains).

### 4.2. RNA Extraction and RNA Sequencing

Total RNA was extracted according to the instructions of RNeasy Plus Mini Kit (Qiagen, Venlo, The Netherlands). A total of 1% agarose gel electrophoresis and NanoDrop 2000 spectrophotometer (Thermo Fisher Scientific, Wilmington, DE, USA) were used to detect the quality and concentration of RNA. An amount of 1 µg of RNA was reversed to cDNA by random hexamer primer (Genstar, Beijing, China). Then, the library was constructed using Illumina platform.

### 4.3. Analysis of RNA Sequencing Data

All data can be searched in the NCBI database with the project number PRJNA1153073. Before data analysis, it is necessary to ensure that these reads are of sufficient quality to ensure the accuracy of subsequent analysis. We carried out strict quality control of the data and performed the following filtering methods: removed reads containing the adaptors and removed low-quality reads (including reads that removed more than 10% of N and removed more than 50% of the reads with mass value Q ≤ 10). The gene’s function was annotated based on the following databases of GO [107] and KEGG [108].

### 4.4. Analysis of Differentially Expressed Genes (DEGs)

The gene expression levels were calculated by the fragment per kilobase of exon per million fragments mapped (FPKMs). The DEGs under different environmental stresses were analyzed using negative binomial distribution of soft DESeq2 [109]. In the process of differential expression gene detection, fold change ≥ 2 and FDR < 0.01 were used as the screening criteria. Fold change represents the ratio of the expression levels between the two samples (groups). The false discovery rate (FDR) was obtained by correcting the *p*-value of the difference significance. The *p*-values were analyzed using the Benjamini and Hochberg approach. Genes with |log_2_FC| ≥ 1 and log_2_FC with *p* < 0.01 were defined as significantly differentially expressed. The volcano map was made with online program chiplot (https://www.chiplot.online/?#BioPlot, accessed on 1 November 2024). The GO and KEGG enrichment analyses of DEGs were performed using the R/clusterProfiler version 3.2 and R/topGO software version 3.2. In order to obtain more differential NPs and NPRs of *S. frugiperda* in response to different environmental pressures, all studies were performed in three biological replicates. The results were plotted with Origin (Northampton, MA, USA). Means were compared using independent sample *t* test, with Type I error = 0.05 as the discriminant threshold. The heat map is described by Origin2021. The RNA-seq data were also analyzed using a PCA in Prism v9.3.1 (GraphPad, San Diego, CA, USA).

### 4.5. Identification of Genes Coding for NPs and Their Receptors and Enzymes Involved in BA Synthesis

The known NP and neuropeptide receptor (NPR) sequences of *S. frugiperda*, *D. melanogaster* and *Bombyx mori* were used as query sequences, and the basic local alignment search tool (tblastn and blastp) was used in the database (https://www.ncbi.nlm.nih.gov/, accessed on 2 October 2024) to search for sequences homologous to the protein sequence of *S. frugiperda*, with an e-value threshold of 10^−5^.

## 5. Conclusions

This project was conducted with the important and invasive *Spodoptera frugiperda* pest, the fall armyworm, and we investigated its transcriptome responses to four different types of stresses, namely cold, heat, no drinking and no food. Here, we used dissected brains as our interest had a focus on the neuroendocrine responses, while previous studies used whole bodies of larvae or moths. In general, the responses were complex and encompassed a vast array of neuropeptides (NPs) and biogenic amines (BAs). The NPs were mainly involved in ion homeostasis regulation (ITP and ITPL) and metabolic pathways (AKH, ILP), and this was accompanied by changes in BA (DA, OA) biosynthesis. Cold stress and no-drinking–water stress altered the same NP gene expression in a similar direction, but the magnitude of these changes was different enough to separate the two stresses from each other. In contrast, no-food stress resulted in the most divergent pattern of NP gene expression. In conclusion, our data present a transcriptome database of *S. frugiperda*, which is an interesting model to study the high adaptive and invasive potential in insects, and in turn, they can provide new targets for controlling important pests. One could target PDF, SIFa or NPF, as these are among the most upregulated NP genes across all stress conditions, indicating their essential roles in stress responses. Silencing one of these genes could potentially impair the insect’s ability to survive under stress conditions. While this may not necessarily result in immediate lethality, it would likely reduce the insect’s overall fitness and resilience, making such a strategy a viable approach for pest management. However, this needs to be proven in laboratory and field conditions.

## Figures and Tables

**Table 1:** Summary statistics of transcriptome sequencing data of the brain samples of *S. frugiperda* under four different stress conditions. Note: Samples = sample analysis number. Clean reads = total number of pair-end reads in clean data. Clean bases = total base number of clean data. GC content = clean data GC content, which is the percentage of G and C bases in clean data. % ≥Q30 = the percentage of bases with a clean data mass value greater than or equal to 30.

Samples	Clean Reads	Clean Bases	GC Content	% ≥Q30
Control 1	39,353,618	11,769,340,976	47.83%	94.52%
Control 2	39,694,672	11,858,613,550	47.98%	94.62%
Control 3	35,381,769	10,570,129,652	47.83%	94.91%
Cold 1	37,717,765	11,269,080,484	51.28%	94.37%
Cold 2	34,513,213	10,316,785,008	49.31%	94.45%
Cold 3	37,715,856	11,280,688,670	50.12%	94.56%
Heat 1	36,763,721	10,983,853,776	48.28%	94.95%
Heat 2	41,152,363	12,299,637,872	47.10%	94.78%
Heat 3	31,884,413	9,526,556,450	48.64%	94.68%
No-water 1	38,897,852	11,626,808,514	49.99%	94.64%
No-water 2	33,478,909	10,011,637,214	50.04%	94.46%
No-water 3	34,041,608	10,174,564,996	48.68%	94.50%
No-food 1	35,476,086	10,605,760,382	48.13%	94.86%
No-food 2	33,775,697	10,102,504,652	48.38%	95.01%
No-food 3	39,602,888	11,840,921,396	48.49%	94.72%

**Table 2:** Statistical table of the number of DEGs (fold change ≥ 2.0 and *p* < 0.01) in the brain samples of *S. frugiperda* under four different stress conditions.

Stress Conditions	Total	#Upregulated	#Downregulated
Cold vs. Control	2448	2024	424
Heat vs. Control	2323	1206	1117
No-water vs. Control	4416	2286	2130
No-food vs. Control	1691	969	722

**Table 3:** The absolute expression (mean ± SEM) of identified neuropeptides (NPs) under control and four tested stress conditions (cold, heat, no water, no food) in the brain of *S. frugiperda*. The absolute expression scalar uses three technically repeated FPKM values and uses the independent sample *t*-test in the software SPSS22.0 to calculate its (mean ± SEM).

Name of Neuropeptide	Gene ID	Control	Cold	Heat	No-Water	No-Food
Adipokinetic Corazonin-related peptide	gene-LOC118277531	0.19 ± 0.03	0.06 ± 0.02	0.19 ± 0.03	0.27 ± 0.07	0.43 ± 0.08
Adipokinetic hormone1	Spodoptera_frugiperda_newGene_1784	1.63 ± 0.66	0.87 ± 0.57	1.30 ± 0.33	2.64 ± 0.66	3.02 ± 0.36
Adipokinetic hormone2	Spodoptera_frugiperda_newGene_1777	0.68 ± 0.07	0.06 ± 0.05	0.69 ± 0.16	0.42 ± 0.18	1.56 ± 0.29
AllatostatinA	gene-LOC118271067	0.66 ± 0.07	0.30 ± 0.16	0.89 ± 0.08	0.55 ± 0.21	1.36 ± 0.18
AllatostatinB	gene-LOC118271332	2.38 ± 0.17	0.77 ± 0.29	2.34 ± 0.10	1.48 ± 0.23	3.78 ± 0.55
Allatotropin	gene-LOC118272809	2.24 ± 0.18	0.76 ± 0.21	2.24 ± 0.11	1.61 ± 0.29	3.74 ± 0.17
AllatostatinC	gene-LOC118269156	1.10 ± 0.13	0.15 ± 0.09	0.81 ± 0.07	0.78 ± 0.16	1.38 ± 0.33
AllatostatinCC	gene-LOC118269242	0.52 ± 0.11	0.18 ± 0.09	0.26 ± 0.03	0.33 ± 0.04	0.75 ± 0.18
Allatotropin like peptide	gene-LOC118272809	2.24 ± 0.18	0.76 ± 0.21	2.24 ± 0.11	1.61 ± 0.29	3.74 ± 0.17
Bursiconalpha	gene-LOC118276337	1.14 ± 0.14	0.25 ± 0.13	0.79 ± 0.21	1.08 ± 0.25	1.19 ± 0.29
Bursicon beta	gene-LOC118276251	1.62 ± 0.29	0.29 ± 0.24	1.17 ± 0.33	1.42 ± 0.20	1.44 ± 0.31
CAPA	gene-LOC118271186	0.52 ± 0.08	0.20 ± 0.11	0.49 ± 0.04	0.40 ± 0.11	0.62 ± 0.04
Calcitonin-B/DH30	gene-LOC118278084	0.03 ± 0.03	0.09 ± 0.05	0.03 ± 0.03	3.30 ± 3.30	0.03 ± 0.03
CCAP	gene-LOC118278715	0.03 ± 0.03	0.02 ± 0.02	0.10 ± 0.07	0.02 ± 0.02	0.13 ± 0.03
CCHamide1	gene-LOC118269812	0.22 ± 0.09	0.07 ± 0.07	0.18 ± 0.10	0.09 ± 0.06	0.31 ± 0.12
CCHamide2	gene-LOC118269803	0.43 ± 0.08	0.29 ± 0.14	0.82 ± 0.04	0.88 ± 0.48	0.87 ± 0.37
Corazonin	gene-LOC118270481	1.17 ± 0.11	0.29 ± 0.11	1.44 ± 0.20	0.84 ± 0.22	2.27 ± 0.25
Diuretic hormone 31	gene-LOC118276433	2.42 ± 0.19	0.86 ± 0.19	2.35 ± 0.24	2.74 ± 0.93	4.13 ± 0.22
Diuretic hormone 34	gene-LOC118262874	5.62 ± 0.12	1.88 ± 0.51	5.10 ± 0.24	3.33 ± 0.38	8.45 ± 1.16
Eclosion hormone	gene-LOC118269902	3.88 ± 1.85	2.30 ± 1.15	0.79 ± 0.25	3.52 ± 0.98	4.80 ± 1.51
Ecdysis triggering hormone	gene-LOC118280022	2.25 ± 0.80	0.40 ± 0.40	0 ± 0	0.30 ± 0.10	0.64 ± 0.42
FMRFamide	gene-LOC118271300	0.58 ± 0.14	0.10 ± 0.07	0.44 ± 0.04	0.36 ± 0.09	0.67 ± 0.19
Glycoprotein hormone alpha 2	gene-LOC118281577	0.09 ± 0.06	0 ± 0	0.01 ± 0.01	0 ± 0	0.08 ± 0.04
Glycoprotein hormone beta5	gene-LOC118265105	0.28 ± 0.07	0.19 ± 0.12	0.27 ± 0.02	0.09 ± 0.05	0.17 ± 0.03
IMFamide	gene-LOC118279669	1.53 ± 0.53	0.38 ± 0.23	1.00 ± 0.16	1.13 ± 0.12	2.33 ± 0.37
Ion-transport peptide	gene-LOC118265982	19.9 ± 1.8	44.6 ± 4.9	17.2 ± 2.1	35.3 ± 4.1	28.2 ± 4.6
ITG	gene-LOC118279990	11.0 ± 0.1	3.78 ± 1.06	12.4 ± 0.5	9.45 ± 1.04	16.1 ± 2.0
Insulin-like peptide1	gene-LOC118263255	2.86 ± 0.66	0.91 ± 0.70	2.19 ± 0.47	1.86 ± 0.24	5.75 ± 2.12
Insulin-like peptide2	gene-LOC118268418	1.22 ± 0.06	0.43 ± 0.18	1.20 ± 0.21	0.85 ± 0.24	1.66 ± 0.42
Insulin-like peptide3	gene-LOC118261854	1.34 ± 0.30	0.26 ± 0.13	1.03 ± 0.40	0.80 ± 0.40	1.01 ± 0.28
Insulin-like peptide4	gene-LOC118271601	0.64 ± 0.07	0.28 ± 0.30	0.82 ± 0.21	0.51 ± 0.21	1.11 ± 0.06
Insulin-like peptide5	gene-LOC118272446	0.20 ± 0.08	0 ± 0	0.06 ± 0.03	0.07 ± 0.07	0.09 ± 0.06
Insulin-like peptide6	gene-LOC118272599	2.02 ± 0.01	0.67 ± 0.14	2.11 ± 0.22	1.14 ± 0.33	3.12 ± 0.19
Kinin	gene-LOC118278057	0.38 ± 0.03	0.18 ± 0.14	0.47 ± 0.10	0.32 ± 0.07	0.62 ± 0.09
LQDVamide	gene-LOC118271314	6.14 ± 0.15	1.95 ± 0.50	6.56 ± 0.13	5.11 ± 0.45	9.99 ± 1.05
Myosupressin	gene-LOC118278224	2.29 ± 0.35	1.15 ± 0.31	2.31 ± 0.29	2.51 ± 0.69	3.42 ± 0.66
Natalisin	gene-LOC118278273	0.73 ± 0.07	0.35 ± 0.06	0.91 ± 0.04	0.64 ± 0.08	1.20 ± 0.11
Neuropeptide F 1a	gene-LOC118269735	0.70 ± 0.02	0.30 ± 0.17	0.71 ± 0.19	0.42 ± 0.12	1.03 ± 0.26
Neuropeptide F 1b	gene-LOC118269652	0.51 ± 0.14	0.16 ± 0.08	0.69 ± 0.10	0.80 ± 0.44	1.11 ± 0.03
Neuropeptide F 2	gene-LOC118279931	0.16 ± 0.02	0.06 ± 0.05	0.33 ± 0.11	0.15 ± 0.09	0.25 ± 0.03
Neuroparsin	Spodoptera_frugiperda_newGene_169	0.53 ± 0.09	0.21 ± 0.01	0.47 ± 0.06	0.25 ± 0.04	0.54 ± 0.03
Neuropeptide-like precursor	gene-LOC118271872	1.60 ± 0.12	0.43 ± 0.16	1.20 ± 0.20	1.14 ± 0.29	2.19 ± 0.38
Orcokinin A	gene-LOC118268956	1.20 ± 0.03	0.45 ± 0.17	0.83 ± 0.10	2.74 ± 1.95	1.33 ± 0.22
PBAN	gene-LOC118281022	3.24 ± 0.41	0.51 ± 0.26	2.53 ± 0.45	2.37 ± 0.21	5.14 ± 0.52
Pigment dispersing factor	gene-LOC118266686	0.03 ± 0.03	0.05 ± 0.03	0.09 ± 0.02	0.07 ± 0.04	0.10 ± 0.06
Prothoracicotropic hormone	gene-LOC118281630	0.41 ± 0.06	0.20 ± 0.07	0.40 ± 0.06	0.41 ± 0.19	0.87 ± 0.29
RYamide	gene-LOC118279919	0.02 ± 0.02	0.01 ± 0.01	0.04 ± 0.02	0.07 ± 0.03	0.06 ± 0.04
Short neuropeptide F	gene-LOC118268318	0.58 ± 0.14	0.34 ± 0.13	0.78 ± 0.13	0.48 ± 0.22	0.70 ± 0.11
Sulfakinin	Spodoptera_frugiperda_newGene_1951	0.66 ± 0.24	0.15 ± 0.07	0.52 ± 0.06	0.25 ± 0.13	0.60 ± 0.10
Tachykinin	gene-LOC118278153	0.64 ± 0.04	0.20 ± 0.12	0.60 ± 0.10	0.81 ± 0.334	0.80 ± 0.17
Trissin-1	gene-LOC118278852	0.61 ± 0.04	0.48 ± 0.10	0.59 ± 0.31	0.62 ± 0.24	1.23 ± 0.15
Trissin-2	gene-LOC118278765	0.15 ± 0.09	0.16 ± 0.13	0.42 ± 0.15	0.26 ± 0.09	0.48 ± 0.12
SIFamide	Spodoptera_frugiperda_newGene_2323	0.03 ± 0.03	0.35 ± 0.18	0.09 ± 0.05	0 ± 0	0.30 ± 0.20

**Table 4:** The absolute expression (mean ± SEM) of identified neuropeptide receptors (NPRs) under control and four tested stress conditions (cold, heat, no water, no food) in the brain of *S. frugiperda*.

Name of Neuropeptide Receptor	Gene ID	Control	Cold	Heat	No-Water	No-Food
ACP Receptor-1	gene-LOC118273818	0.03 ± 0.01	0.01 ± 0.01	0.05 ± 0.03	0.03 ± 0.02	0.02 ± 0.02
ACP Receptor-2	gene-LOC118274328	0.01 ± 0.01	0 ± 0	0 ± 0	0.02 ± 0.02	0.04 ± 0.04
AKH Receptor	gene-LOC118265813	1.48 ± 0.37	0.22 ± 0.09	0.30 ± 0.07	0.28 ± 0.11	0.29 ± 0.06
AST-A Receptor	gene-LOC118264343	0.76 ± 0.14	0.37 ± 0.10	0.48 ± 0.08	0.50 ± 0.10	0.80 ± 0.07
AST-C Receptor	gene-LOC118266942	0.81 ± 0.05	0.71 ± 0.23	0.47 ± 0.06	0.82 ± 0.24	0.71 ± 0.02
Bursicon Receptor	gene-LOC118271105	3.70 ± 0.42	3.02 ± 0.34	7.25 ± 0.66	2.24 ± 0.30	1.79 ± 0.36
CAPA-PVK Receptor-1	gene-LOC118271027	0.04 ± 0.02	0.09 ± 0.02	0.11 ± 0.05	0.07 ± 0.04	0.04 ± 0.02
CAPA-PVK Receptor-2	gene-LOC118271188	0.07 ± 0.03	0.01 ± 0.01	0.03 ± 0.01	0 ± 0	0.05 ± 0.01
CCAP Receptor-1	gene-LOC118280624	1.49 ± 0.09	1.73 ± 0.17	1.09 ± 0.18	1.01 ± 0.09	1.11 ± 0.16
CCAP Receptor-2	gene-LOC118261782	0.06 ± 0.02	0.01 ± 0.01	0.04 ± 0	0.02 ± 0.01	0.02 ± 0.02
CCHa1 Receptor-1	gene-LOC118271772	0.05 ± 0.01	0.03 ± 0.02	0.05 ± 0.01	0.02 ± 0.02	0.06 ± 0.03
CCHa2 Receptor	gene-LOC118271882	1.31 ± 0.09	0.75 ± 0.25	0.99 ± 0.05	1.48 ± 0.31	1.31 ± 0.09
CNMamide Receptor	gene-LOC118274298	0.02 ± 0.01	0 ± 0	0.01 ± 0.01	0.01 ± 0	0.01 ± 0.01
Corazonin Receptor	gene-LOC118282009	0.32 ± 0.03	0.22 ± 0.05	0.09 ± 0.03	0.39 ± 0.21	0.21 ± 0.07
DH31/Calcitonin receptor-1	gene-LOC118268437	0.35 ± 0.09	1.00 ± 0.15	0.09 ± 0.03	1.22 ± 0.72	0.75 ± 0.09
DH31/Calcitonin receptor-2	gene-LOC118268743	0.98 ± 0.12	1.08 ± 0.08	0.70 ± 0.10	1.11 ± 0.09	1.31 ± 0.13
DH44 Receptor-1	gene-LOC118265921	1.03 ± 0.11	1.09 ± 0.20	0.55 ± 0.15	0.91 ± 0.11	0.87 ± 0.03
DH44 Receptor-2	gene-LOC118265919	1.32 ± 0.11	1.11 ± 0.15	0.64 ± 0.11	0.99 ± 0.17	0.95 ± 0.16
ETH Receptor	gene-LOC118270299	1.04 ± 0.14	0.34 ± 0.11	0.66 ± 0.08	0.57 ± 0.07	0.62 ± 0.08
EH Receptor	gene-LOC118267004	1.35 ± 0.09	0.74 ± 0.20	1.05 ± 0.07	0.60 ± 0.11	0.66 ± 0.06
FMRFa Receptor	gene-LOC118262547	14.4 ± 0.9	7.23 ± 1.73	12.2 ± 1.0	16.1 ± 1.7	23.2 ± 1.2
GPA2/GPA5 Receptor	gene-LOC118269334	0.91 ± 0.11	0.90 ± 0.32	0.73 ± 0.01	1.05 ± 0.11	0.95 ± 0.13
Insulin Receptor-1	gene-LOC118271105	3.70 ± 0.42	3.02 ± 0.34	7.25 ± 0.66	2.24 ± 0.30	1.79 ± 0.36
Insulin Receptor-2	gene-LOC118271704	3.08 ± 0.20	2.14 ± 0.40	4.16 ± 0.18	3.78 ± 0.50	6.16 ± 0.52
Insulin Receptor-3	gene-LOC118270523	3.52 ± 0.20	2.02 ± 0.28	3.80 ± 0.08	3.76 ± 0.61	5.75 ± 0.25
ITP receptor	gene-LOC118271902	0 ± 0	0.02 ± 0.02	0.05 ± 0.02	0.01 ± 0.01	0 ± 0
Kinin Receptor	gene-LOC118261869	0.21 ± 0.02	0.39 ± 0.13	0.17 ± 0.01	0.13 ± 0.03	0.24 ± 0.07
MS Receptor-1	gene-LOC118266966	0.68 ± 0.09	1.41 ± 0.18	0.33 ± 0.06	1.13 ± 0.04	0.55 ± 0.03
MS Receptor-2	gene-LOC118267191	1.59 ± 0.07	1.99 ± 0.09	0.71 ± 0.11	1.69 ± 0.15	1.91 ± 0.09
NPF Receptor	gene-LOC118270649	0.08 ± 0.02	0.12 ± 0.04	0.08 ± 0.01	0.18 ± 0.05	0.06 ± 0.01
NTL Receptor1	gene-LOC118268412	0.06 ± 0.03	0.02 ± 0.02	0.12 ± 0.05	0.04 ± 0.02	0.06 ± 0.03
NTL Receptor-2	gene-LOC118269913	0.21 ± 0.01	0.03 ± 0.02	0.11 ± 0.02	0.07 ± 0.01	0.09 ± 0
NPLP Receptor	gene-LOC118267003	11.1 ± 0.3	8.21 ± 0.74	11.3 ± 0.3	8.99 ± 0.51	12.2 ± 0.4
PDF Receptor	gene-LOC118265406	0.08 ± 0.02	0.02 ± 0.01	0.06 ± 0.01	0.03 ± 0.02	0.08 ± 0.02
pyrokinin-1 Receptor	gene-LOC118264923	2.79 ± 0.18	6.89 ± 1.40	1.01 ± 0.20	4.73 ± 0.08	3.39 ± 0.49
PTTH Receptor	gene-LOC118266498	5.67 ± 0.66	4.45 ± 0.50	3.73 ± 0.23	2.48 ± 0.19	4.58 ± 0.60
PABN Receptor	gene-LOC118264924	2.55 ± 0.19	3.26 ± 0.19	1.83 ± 0.16	3.13 ± 0.21	2.60 ± 0.31
Ryamide Receptor-1	gene-LOC118268644	0.26 ± 0.07	0.10 ± 0.01	0.15 ± 0.03	0.11 ± 0.02	0.20 ± 0.04
Ryamide Receptor-2	gene-LOC118268638	0.61 ± 0.13	0.18 ± 0.06	0.24 ± 0.09	0.28 ± 0.05	0.18 ± 0.05
Sex peptide Receptor	gene-LOC118276693	0.09 ± 0.01	0.28 ± 0.01	0.15 ± 0.03	0.19 ± 0.07	0.14 ± 0.01
sNPF Receptor-1	gene-LOC118272572	0.19 ± 0.03	0.13 ± 0.04	0.20 ± 0.08	0.12 ± 0.02	0.26 ± 0.02
sNPF Receptor-2	gene-LOC118272566	0.07 ± 0.01	0.03 ± 0.01	0.05 ± 0	0.08 ± 0.02	0.09 ± 0.01
sNPF Receptor-3	gene-LOC118272588	2.00 ± 0.11	0.69 ± 0.13	1.86 ± 0.17	1.04 ± 0.19	1.33 ± 0.20
Sulfakinin Receptor	gene-LOC118263811	0.22 ± 0.19	0.01 ± 0.01	0.36 ± 0.11	0.22 ± 0.19	0.35 ± 0.14
SIFamide Receptor	gene-LOC118269799	0.10 ± 0.02	0.72 ± 0.17	0.13 ± 0.03	0.32 ± 0.02	0.06 ± 0.03
Tachykinin Receptor	gene-LOC118281565	0.39 ± 0.01	0.27 ± 0.06	0.43 ± 0.02	0.62 ± 0.14	0.81 ± 0.04
Trissin Receptor	gene-LOC118273356	0.81 ± 0.08	0.67 ± 0.21	0.52 ± 0.17	0.72 ± 0.08	1.02 ± 0.06
Orphan Receptor-1	gene-LOC118277097	0.06 ± 0.01	0.04 ± 0.02	0.03 ± 0.02	0.08 ± 0.02	0.05 ± 0.03
Orphan Receptor-2	gene-LOC118277095	0.07 ± 0.03	0.12 ± 0.04	0.06 ± 0.05	0.08 ± 0.01	0.12 ± 0.03
Orphan Receptor-3	gene-LOC118282249	0.07 ± 0.02	0.08 ± 0.01	0.08 ± 0.02	0.09 ± 0.03	0.12 ± 0.01
Orphan Receptor-4	gene-LOC118266511	0.07 ± 0.02	0.08 ± 0.03	0.02 ± 0.01	0.08 ± 0.03	0.24 ± 0.02
Orphan Receptor-5	gene-LOC118266367	0.17 ± 0.04	0.04 ± 0.03	0.11 ± 0.03	0.11 ± 0.01	0.22 ± 0.04
Orphan Receptor-6	gene-LOC118266407	0.18 ± 0.08	0.14 ± 0.01	0.05 ± 0.02	0.25 ± 0.05	0.33 ± 0.03
Orphan Receptor-7	gene-LOC118267191	1.59 ± 0.07	1.99 ± 0.09	0.71 ± 0.11	1.69 ± 0.15	1.91 ± 0.09
Orphan Receptor-8	gene-LOC118267704	3.31 ± 0.35	6.16 ± 0.62	3.78 ± 0.07	5.08 ± 0.09	3.19 ± 0.36
Orphan Receptor-9	gene-LOC118274442	0.24 ± 0.04	0.30 ± 0.27	1.05 ± 0.43	0.18 ± 0.07	0.18 ± 0.03
Orphan Receptor-10	gene-LOC118265380	0 ± 0	0 ± 0	0.02 ± 0.01	0.02 ± 0.01	0.01 ± 0.01

**Table 5:** The absolute expression (mean ± SEM) of genes encoding biogenic amine (BA) synthesis enzymes under control and four tested stress conditions (cold, heat, no-water, no-food) in the brain of *S. frugiperda*.

Name of Enzyme	Gene ID	Control	Cold	Heat	No-Water	No-Food
Tyramine beta hydroxylase (TβH)	gene-LOC118281786	5.58 ± 0.45	1.43 ± 0.56	1.01 ± 0.20	0.96 ± 0.38	2.77 ± 0.41
Aromatic-L-amino-acid decarboxylase (DDC)	gene-LOC118272996	28 ± 8	614 ± 5	29 ± 2	401 ± 88	50 ± 8
Histidine decarboxylase (HDC)	gene-LOC118279687	0.16 ± 0.06	0.26 ± 0.24	0.40 ± 0.28	0.53 ± 0.24	1.37 ± 0.33
Tryptophan 5-hydroxylase (TPH)	gene-LOC118262869	0.97 ± 0.12	0.19 ± 0.21	1.10 ± 0.22	0.55 ± 0.07	1.27 ± 0.10
Tyrosine decarboxylase (TDC)	gene-LOC118267010	0.42 ± 0.05	0.14 ± 0.13	0.38 ± 0.12	0.31 ± 0.06	0.47 ± 0.06
Tyrosine hydroxylase (TH)	gene-LOC118263443	464 ± 182	5221 ± 21	83 ± 5	2740 ± 525	467 ± 193

## Data Availability

The original contributions presented in this study are included in the article/Supplementary Materials. Further inquiries can be directed to the corresponding author.

## Supplementary Materials

The following supporting information can be downloaded at: https://www.mdpi.com/article/10.3390/ijms26020691/s1.

## References

[1] Lubawy J., Urbański A., Colinet H., Pflüger H.J., Marciniak P. (2020). Role of the insect neuroendocrine system in the response to cold stress. Front. Physiol..

[2] Sinclair B.J., Ferguson L.V., Salehipour-Shirazi G., MacMillan H.A. (2013). Cross-tolerance and cross-talk in the cold: Relating low temperatures to desiccation and immune stress in insects. Integr. Comp. Biol..

[3] El-Saadi M.I., MacMillan H.A., Ferguson L.V. (2023). Cold-induced immune activation in chill-susceptible insects. Curr. Opin. Insect Sci..

[4] Overgaard J., MacMillan H.A. (2017). The integrative physiology of insect chill tolerance. Annu. Rev. Physiol..

[5] Bodlah M.A., Iqbal J., Ashiq A., Bodlah I., Jiang S., Mudassir M.A., Rasheed M.T., Fareen A.G.E. (2023). Insect behavioral restraint and adaptation strategies under heat stress: An inclusive review. J. Saudi Soc. Agric. Sci..

[6] Schoofs L., De Loof A., Van Hiel M.B. (2017). Neuropeptides as regulators of behavior in insects. Annu. Rev. Entomol..

[7] Lubawy J., Chowański S., Adamski Z., Słocińska M. (2022). Mitochondria as a target and central hub of energy division during cold stress in insects. Front. Zool..

[8] Gallio M., Ofstad T.A., Macpherson L.J., Wang J.W., Zuker C.S. (2011). The coding of temperature in the *Drosophila* brain. Cell.

[9] Robertson R.M. (2004). Thermal stress and neural function: Adaptive mechanisms in insect model systems. J. Therm. Biol..

[10] Sparks A.N. (1979). A review of the biology of the fall armyworm. Florida Entomol..

[11] Montezano D.G., Sosa-Gómez D.R., Specht A., Roque-Specht V.F., Sousa-Silva J.C., Paula-Moraes S.V., Peterson J.A., Hunt T.E. (2018). Host plants of *Spodoptera frugiperda* (Lepidoptera: Noctuidae) in the Americas. Afr. Entomol..

[12] Vatanparast M., Park Y. (2022). Differential transcriptome analysis reveals genes related to low and high-temperature stress in the fall armyworm, *Spodoptera frugiperda*. Front. Physiol..

[13] Yang C.-L., Meng J.-Y., Zhou J.-Y., Zhang J.-S., Zhang C.-Y. (2024). Integrated transcriptomic and proteomic analyses reveal the molecular mechanism underlying the thermotolerant response of *Spodoptera frugiperda*. Int. J. Biol. Macromol..

[14] Gokulanathan A., Mo H.-H., Park Y. (2024). Glucose influence cold tolerance in the fall armyworm, *Spodoptera frugiperda* via trehalase gene expression. Sci. Rep..

[15] Tao Y.-D., Liu Y., Wan X.-S., Xu J., Fu D.-Y., Zhang J.-Z. (2023). High and low temperatures differentially affect survival, reproduction, and gene transcription in male and female moths of *Spodoptera frugiperda*. Insects.

[16] Vatanparast M., Park Y. (2022). Cold tolerance strategies of the fall armyworm, *Spodoptera frugiperda* (Smith) (Lepidoptera: Noctuidae). Sci. Rep..

[17] Yan X., Zhao Z., Feng S., Zhang Y., Wang Z., Li Z. (2024). Multi-omics analysis reveal the fall armyworm *Spodoptera frugiperda* tolerate high temperature by mediating chitin-related genes. Insect Biochem. Mol. Biol..

[18] Chen X., Tan A., Palli S.R. (2020). Identification and functional analysis of promoters of heat-shock genes from the fall armyworm, *Spodoptera frugiperda*. Sci. Rep..

[19] Zhou L., Meng J.-Y., Ruan H.-Y., Zhang C.-Y. (2023). Expression analysis of *HSP70* gene in response to environmental stress in *Spodoptera frugiperda* (Lepidoptera: Noctuidae). J. Asia-Pac. Entomol..

[20] Gao X., Lin Y., Zhang Z., Qiu L., Dong W., Gao Q., Gao H., Xue J., Li Y., He H. (2024). Storage protein *SfSP8* mediates larval starvation tolerance of *Spodoptera frugiperda*. Mol. Biol. Rep..

[21] Xu H.M., Zhao H.Z., Pan M.Z., Smagghe G., Li Z.Y., Liu T.X., Shi Y. (2023). Regulating role of neuropeptide PTTH releaved in *Spodoptera frugiperda* using RNAi-and CRISPR/Cas9-based functional genomic tools. Entomol. Gen..

[22] Wagner D.L., Grames E.M., Forister M.L., Berenbaum M.R., Stopak D. (2021). Insect decline in the Anthropocene: Death by a thousand cuts. Proc. Natl. Acad. Sci. USA.

[23] Fields P., Subramanyam B., Hulasare R., Hagstrum D.W., Phillips T.W., Cuperus G.W. (2012). Extreme Temperatures, Stored Product Protection.

[24] Bale J.S., Hayward S.A.L. (2010). Insect overwintering in a changing climate. J. Exp. Biol..

[25] Enriquez T., Colinet H. (2019). Cold acclimation triggers lipidomic and metabolic adjustments in the spotted wing drosophila *Drosophila suzukii* (Matsumara). Am. J. Physiol. Regul. Integr. Comp. Physiol..

[26] Lubawy J., Hornik J. (2022). The effect of B-type allatostatin neuropeptides on crosstalk between the insect immune response and cold tolerance. Sci. Rep..

[27] Svensson O., Arnebrant T. (2010). Adsorption of serum albumin on silica–The influence of surface cleaning procedures. J. Coll. Interface Sci..

[28] Wang Z., Zhou J., Li J., Zou J., Fan L. (2020). The immune defense response of Pacific white shrimp (*Litopenaeus vannamei*) to temperature fluctuation. Fish Shellfish Immunol..

[29] Findsen A., Pedersen T.H., Petersen A.G., Nielsen O.B., Overgaard J. (2014). Why do insects enter and recover from chill coma? Low temperature and high extracellular potassium compromise muscle function in *Locusta migratoria*. J. Exp. Biol..

[30] Armstrong G.A., Rodríguez E.C., Robertson R.M. (2012). Cold hardening modulates K+ homeostasis in the brain of *Drosophila melanogaster* during chill coma. J. Insect Physiol..

[31] Rodgers C.I., Armstrong G.A., Robertson R.M. (2010). Coma in response to environmental stress in the locust: A model for cortical spreading depression. J. Insect Physiol..

[32] Edney E.B. (2012). Water Balance in Land Arthropods.

[33] Sun L., Zhang Z., Zhang R., Yu Y., Yang F., Tan A. (2020). Molecular disruption of ion transport peptide receptor results in impaired water homeostasis and developmental defects in *Bombyx mori*. Front. Physiol..

[34] Zandawala M. (2012). Calcitonin-like diuretic hormones in insects. Insect Biochem. Mol. Biol..

[35] Roller L., Čižmár D., Gáliková Z., Bednár B., Daubnerová I., Žitňan D. (2016). Molecular cloning, expression and identification of the promoter regulatory region for the neuropeptide trissin in the nervous system of the silkmoth *Bombyx mori*. Cell Tissue Res..

[36] Barber A.F., Sehgal A. (2018). Cold temperatures fire up circadian neurons. Cell Metab..

[37] Parker D.J., Envall T., Ritchie M.G., Kankare M. (2021). Sex-specific responses to cold in a very cold-tolerant, northern *Drosophila* species. Heredity.

[38] Grima B., Chélot E., Xia R., Rouyer F. (2004). Morning and evening peaks of activity rely on different clock neurons of the *Drosophila* brain. Nature.

[39] Yoshii T., Hermann-Luibl C., Kistenpfennig C., Schmid B., Tomioka K., Helfrich-Förster C. (2015). Cryptochrome-dependent and-independent circadian entrainment circuits in *Drosophila*. J. Neurosci..

[40] Yao Z., Shafer O.T. (2014). The *Drosophila* circadian clock is a variably coupled network of multiple peptidergic units. Science.

[41] Liang X., Holy T.E., Taghert P.H. (2016). Synchronous *Drosophila* circadian pacemakers display nonsynchronous Ca^2+^ rhythms in vivo. Science.

[42] Nelson M.D., Lee K.H., Churgin M.A., Hill A.J., Van Buskirk C., Fang-Yen C., Raizen D.M. (2014). FMRFamide-like FLP-13 neuropeptides promote quiescence following heat stress in *Caenorhabditis elegans*. Curr. Biol..

[43] Srithiphaphirom P., Lavallee S., Robertson R.M. (2019). Rapid cold hardening and octopamine modulate chill tolerance in *Locusta migratoria*. Comp. Biochem. Physiol. A Mol. Integr. Physiol..

[44] Chen X., Xiao D., Du X., Guo X., Zhang F., Desneux N., Zang L., Wang S. (2019). The role of the dopamine melanin pathway in the ontogeny of elytral melanization in Harmonia axyridis. Front. Physiol..

[45] Zhang Y., Wang X.X., Tian H.G., Zhang Z.F., Feng Z.J., Chen Z.S., Liu T.X. (2020). The L-DOPA/dopamine pathway transgenerationally regulates cuticular melanization in the pea aphid *Acyrthosiphon pisum*. Front. Cell Dev. Biol..

[46] Fedorka K.M., Copeland E.K., Winterhalter W.E. (2013). Seasonality influences cuticle melanization and immune defense in a cricket: Support for a temperature-dependent immune investment hypothesis in insects. J. Exp. Biol..

[47] Urbański A., Adamski Z., Rosiński G. (2018). Developmental changes in haemocyte morphology in response to *Staphylococcus aureus* and latex beads in the beetle *Tenebrio molitor* L.. Micron.

[48] Tong R., Wei C., Pan L., Zhang X. (2020). Effects of dopamine on immune signaling pathway factors, phagocytosis and exocytosis in hemocytes of *Litopenaeus vannamei*. Dev. Comp. Immunol..

[49] Cabrero P., Radford J.C., Broderick K.E., Costes L., Veenstra J.A., Spana E.P., Davies S.A., Dow J.A.T. (2002). The DH gene of Drosophila melanogaster encodes a diuretic peptide that acts through cyclic AMP. J. Exp. Biol..

[50] Cannell E., Dornan A.J., Halberg K.A., Terhzaz S., Dow J.A., Davies S.A. (2016). The corticotropin-releasing factor-like diuretic hormone 44 (DH44) and kinin neuropeptides modulate desiccation and starvation tolerance in *Drosophila melanogaster*. Peptides.

[51] Honer M., Buscemi K., Barrett N., Riazati N., Orlando G., Nelson M.D. (2020). Orcokinin neuropeptides regulate sleep in *Caenorhabditis elegans*. J. Neurogenet..

[52] Jiang H., Kim H.G., Park Y. (2015). Alternatively spliced orcokinin isoforms and their functions in *Tribolium castaneum*. Insect Biochem. Mol. Biol..

[53] Lenz O., Xiong J., Nelson M.D., Raizen D.M., Williams J.A. (2015). FMRFamide signaling promotes stress-induced sleep in *Drosophila*. Brain Behav. Immun..

[54] Gillooly J.F., Brown J.H., West G.B., Savage V.M., Charnov E.L. (2001). Effects of size and temperature on metabolic rate. Science.

[55] González-Tokman D., Córdoba-Aguilar A., Dáttilo W., Lira-Noriega A., Sánchez-Guillén R.A., Villalobos F. (2020). Insect responses to heat: Physiological mechanisms, evolution and ecological implications in a warming world. Biol. Rev..

[56] Pörtner H.O. (2002). Climate variations and the physiological basis of temperature dependent biogeography: Systemic to molecular hierarchy of thermal tolerance in animals. Comp. Biochem. Physiol. A Mol. Integr. Physiol..

[57] Verberk W.C.E.P., Overgaard J., Ern R., Bayley M., Wang T., Boardman L., Terblanche J.S. (2016). Does oxygen limit thermal tolerance in arthropods? A critical review of current evidence. Comp. Biochem. Physiol. A Mol. Integr. Physiol..

[58] Cholewiński M., Chowański S., Lubawy J., Urbański A., Walkowiak-Nowicka K., Marciniak P. (2024). Short neuropeptide F in integrated insect physiology. J. Zhejiang Univ. Sci. B.

[59] Fadda M., Hasakiogullari I., Temmerman L., Beets I., Zels S., Schoofs L. (2019). Regulation of feeding and metabolism by neuropeptide F and short neuropeptide F in invertebrates. Front. Endocrinol..

[60] Martelli C., Pech U., Kobbenbring S., Pauls D., Bahl B., Sommer M.V., Pooryasin A., Barth J., Arias C.W.P., Vassiliou C. (2017). SIFamide translates hunger signals into appetitive and feeding behavior in Drosophila. Cell Rep..

[61] Zdárek J., Nachman R.J., Denlinger D.L. (2000). Parturition hormone in the tsetse *Glossina morsitans*: Activity in reproductive tissues from other species and response of tsetse to identified neuropeptides and other neuroactive compounds. J. Insect Physiol..

[62] Zhu Z., Tsuchimoto M., Nagata S. (2022). CCHamide-2 signaling regulates food intake and metabolism in *Gryllus bimaculatus*. Insects.

[63] Ida T., Takahashi T., Tominaga H., Sato T., Sano H., Kume K., Ozaki M., Hiraguchi T., Shiotani H., Terajima S. (2012). Isolation of the bioactive peptides CCHamide-1 and CCHamide-2 from *Drosophila* and their putative role in appetite regulation as ligands for G protein-coupled receptors. Front. Endocrinol..

[64] Farhan A., Gulati J., Groβe-Wilde E., Vogel H., Hansson B.S., Knaden M. (2013). The CCHamide 1 receptor modulates sensory perception and olfactory behavior in starved *Drosophila*. Sci. Rep..

[65] Fujiwara Y., Hermann-Luibl C., Katsura M., Sekiguchi M., Ida T., Helfrich-Förster C., Yoshii T. (2018). The CCHamide1 neuropeptide expressed in the anterior dorsal neuron 1 conveys a circadian signal to the ventral lateral neurons in *Drosophila melanogaster*. Front. Physiol..

[66] Shi Y., Liu T.Y., Jiang H.B., Liu X.Q., Dou W., Park Y., Smagghe G., Wang J.J. (2019). The ecdysis triggering hormone system, via ETH/ETHR-B, is essential for successful reproduction of a major pest insect, *Bactrocera dorsalis* (Hendel). Front. Physiol..

[67] Van de Velde S., Badisco L., Marchal E., Broeck J.V., Smagghe G. (2009). Diversity in factors regulating ecdysteroidogenesis in insects. Ecdysone: Structures and Functions.

[68] Iga M., Smagghe G. (2010). Identification and expression profile of Halloween genes involved in ecdysteroid biosynthesis in *Spodoptera littoralis*. Peptides.

[69] Bobrovskikh M.A., Gruntenko N.E. (2023). Mechanisms of neuroendocrine Stress Response in *Drosophila* and its effect on carbohydrate and lipid metabolism. Insects.

[70] Keosentse O., Mutamiswa R., Nyamukondiwa C. (2022). Interaction effects of desiccation and temperature stress resistance across *Spodoptera frugiperda* (Lepidoptera, Noctuidae) developmental stages. NeoBiota.

[71] Benoit J.B., McCluney K.E., DeGennaro M.J., Dow J.A. (2023). Dehydration dynamics in terrestrial arthropods: From water sensing to trophic interactions. Annu. Rev. Entomol..

[72] Roggiani M., Srujana S., Yadavalli S.S., Mark Goulian M. (2017). Natural variation of a sensor kinase controlling a conserved stress response pathway in *Escherichia coli*. PLoS Genet..

[73] Hermann-Luibl C., Yoshii T., Senthilan P.R., Dircksen H., Helfrich-Förster C. (2014). The ion transport peptide is a new functional clock neuropeptide in the fruit fly *Drosophila melanogaster*. J. Neurosci..

[74] Chen T., Zhang M., Ding Z., Hu J., Yang J., He L., Jia J., Yang J., Yang J., Song X. (2023). The Drosophila NPY-like system protects against chronic stress–induced learning deficit by preventing the disruption of autophagic flux. Proc. Natl. Acad. Sci. USA.

[75] Bai T.T., Pei X.J., Liu T.X., Fan Y.L., Zhang S.Z. (2022). Melanin synthesis genes BgTH and BgDdc affect body color and cuticle permeability in *Blattella germanica*. Insect Sci..

[76] Castrillon G., Epp S., Bose A., Fraticelli L., Hechler A., Belenya R., Ranft A., Yakushev I., Utz L., Sundar L. (2023). An energy costly architecture of neuromodulators for human brain evolution and cognition. Sci. Adv..

[77] Li Y., Wang X., Dong H., Xia Q., Zhao P. (2023). Transcriptomic analysis of starvation on the silkworm brain. Insects.

[78] Nelson J.M., Saunders C.J., Johnson E.C. (2021). The intrinsic nutrient sensing adipokinetic hormone producing cells function in modulation of metabolism, activity, and stress. Int. J. Mol. Sci..

[79] Goda T., Umezaki Y., Alwattari F., Seo H.W., Hamada F.N. (2019). Neuropeptides PDF and DH31 hierarchically regulate free-running rhythmicity in *Drosophila* circadian locomotor activity. Sci. Rep..

[80] Krashes M.J., DasGupta S., Vreede A., White B., Armstrong J.D., Waddell S. (2009). A neural circuit mechanism integrating motivational state with memory expression in *Drosophila*. Cell.

[81] Inagaki H.K., De-Leon S.B., Wong A.M., Jagadish S., Ishimoto H., Barnea G., Kitamoto T., Axel R., Anderson D.J. (2012). Visualizing neuromodulation in vivo: TANGO-mapping of dopamine signaling reveals appetite control of sugar sensing. Cell.

[82] Damrau C., Toshima N., Tanimura T., Brembs B., Colomb J. (2018). Octopamine and tyramine contribute separately to the counter-regulatory response to sugar deficit in *Drosophila*. Front. Syst. Neurosci..

[83] Selcho M., Pauls D. (2019). Linking physiological processes and feeding behaviors by octopamine. Curr. Opin. Insect Sci..

[84] Nässel D.R., Zandawala M. (2019). Recent advances in neuropeptide signaling in *Drosophila*, from genes to physiology and behavior. Prog. Neurobiol..

[85] Zhong Y., Wang H., Lu G., Zhang Z., Jiao Q., Liu Y. (2009). Detecting functional connectivity in fMRI using PCA and regression analysis. Brain Topogr..

[86] McLachlan G.J., Do K.A., Ambroise C. (2005). Analyzing Microarray Gene Expression Data.

[87] King A.M., MacRae T.H. (2015). Insect heat shock proteins during stress and diapause. Annu. Rev. Entomol..

[88] Storey K.B., Storey J.M. (2004). Metabolic rate depression in animals: Transcriptional and translational controls. Biol. Rev..

[89] Andrew S.C., Hurley L.L., Mariette M.M., Griffith S.C. (2017). Higher temperatures during development reduce body size in the zebra finch in the laboratory and in the wild. J. Evol. Biol..

[90] Ayres J.S., Schneider D.S. (2009). The role of anorexia in resistance and tolerance to infections in *Drosophila*. PLoS Biol..

[91] Abram P.K., Boivin G., Moiroux J., Brodeur J. (2017). Behavioural effects of temperature on ectothermic animals: Unifying thermal physiology and behavioural plasticity. Biol. Rev..

[92] Eremina M.A., Menshanov P.N., Shishkina O.D., Gruntenko N.E. (2021). The transcription factor dfoxo controls the expression of insulin pathway genes and lipids content under heat stress in *Drosophila melanogaster*. Vavilov J. Genet. Breed..

[93] Ugrankar R., Theodoropoulos P., Akdemir F., Henne W.M., Graff J.M. (2018). Circulating glucose levels inversely correlate with *Drosophila* larval feeding through insulin signaling and SLC5A11. Commun. Biol..

[94] Jena K., Kar P.K., Kausar Z., Babu C.S. (2013). Effects of temperature on modulation of oxidative stress and antioxidant defenses in testes of tropical tasar silkworm *Antheraea mylitta*. J. Ther. Biol..

[95] Gérard M., Martinet B., Maebe K., Marshall L., Smagghe G., Vereecken N.J., Vray S., Rasmont P., Michez D. (2020). Shift in size of bumblebee queens over the last century. Glob. Change Biol..

[96] Sehnal F., Sutherland T. (2008). Silks produced by insect labial glands. Prion.

[97] Alonso C., Herrera C.M. (2000). Seasonal variation in leaf characteristics and food selection by larval noctuids on an evergreen Mediterranean shrub. Acta Oecol..

[98] Rodrigues D., Moreira G.R. (2004). Seasonal variation in larval host plants and consequences for *Heliconius erato* (Lepidoptera: Nymphalidae) adult body size. Austral Ecol..

[99] Harrington G.J. (2001). Impact of Paleocene/Eocene greenhouse warming on North American paratropical forests. Palaios.

[100] Root T.L., Price J.T., Hall K.R., Schneider S.H., Rosenzweig C., Pounds J.A. (2003). Fingerprints of global warming on wild animals and plants. Nature.

[101] Battisti A., Stastny M., Buffo E., Larsson S. (2006). A rapid altitudinal range expansion in the pine processionary moth produced by the 2003 climatic anomaly. Glob. Change Biol..

[102] Braschler B., Hill J.K. (2007). Role of larval host plants in the climate-driven range expansion of the butterfly *Polygonia c-album*. J. Anim. Ecol..

[103] Calabrese E.J. (2014). Hormesis: A fundamental concept in biology. Microb. Cell.

[104] Guedes R.N.C., Rix R.R., Cutler G.C. (2022). Pesticide-induced hormesis in arthropods: Towards biological systems. Curr. Opin. Toxicol..

[105] Guedes R.N.C., Smagghe G., Stark J.D., Desneux N. (2016). Pesticide-induced stress in arthropod pests for optimized integrated pest management programs. Annu. Rev. Entomol..

[106] Wu L.H., Cao Z., Long G., Yang X., Wei Z., Liao Y., Hong Y., Hu C. (2021). Fitness of fall armyworm, *Spodoptera frugiperda* to three *Solanaceous* vegetables. J. Integr. Agric..

[107] Ashburner M., Ball C.A., Blake J.A., Botstein D., Butler H., Cherry J.M., Davis A.P., Dolinski K., Dwight S.S., Eppig J.T. (2000). Gene ontology: Tool for the unification of biology. Nat. Genet..

[108] Kanehisa M., Goto S., Kawashima S., Okuno Y., Hattori M. (2004). The KEGG resource for deciphering the genome. Nucleic Acids Res..

[109] Love M.I., Huber W., Anders S. (2014). Moderated estimation of fold change and dispersion for RNA-seq data with DESeq2. Genome biol..

