# Supplementary material for: Transcriptome and Neuroendocrinome Responses to Environmental Stress in the Model and Pest Insect Spodoptera frugiperda
Source: Int J Mol Sci. 2025 Jan 15;26(2):691. doi: 10.3390/ijms26020691 (PMC11766081; doi:10.3390/ijms26020691)
Supplement: Supplementary file 1 [file ijms-26-00691-s001.zip › ijms-3345953-supplementary.pdf]

**Supplementary data**

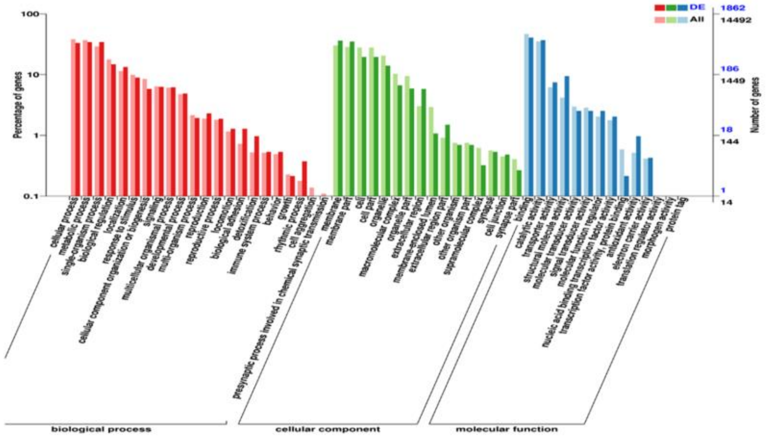


Control vs. Cold

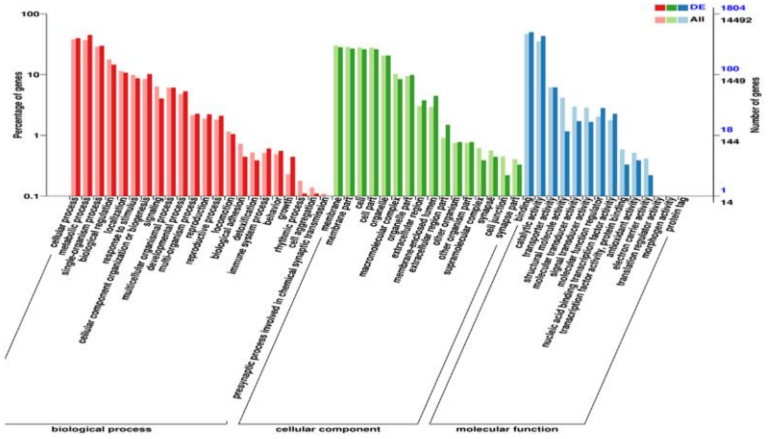


Control vs. Heat

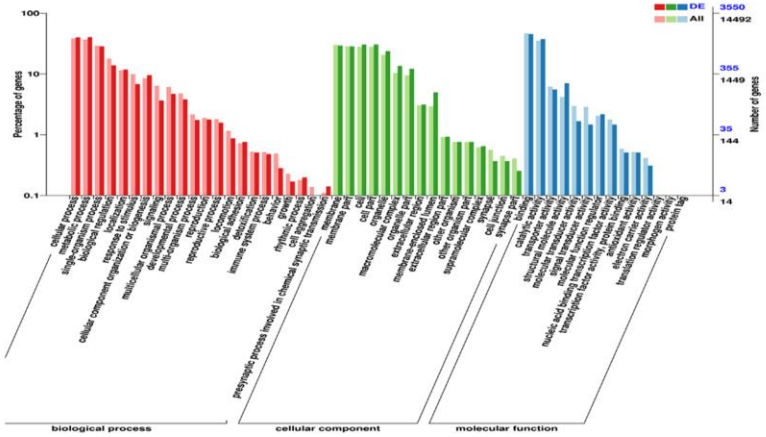


Control vs. No-water

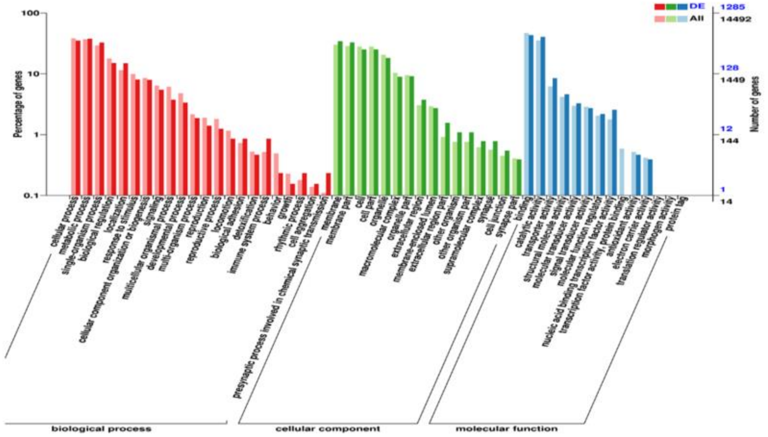


Control vs. No-food

**Figure S1.** Gene Ontology (GO) analysis. GO annotation was performed on the DEGs (P<0.05, log2FC ≥2, ≤-2) in the brain samples of S. *frugiperda* under four different stress conditions. GO items were classified into three main categories: "biological process", "cellular component" and "molecular function". The Y axis represents the percentage of unigenes in each category.

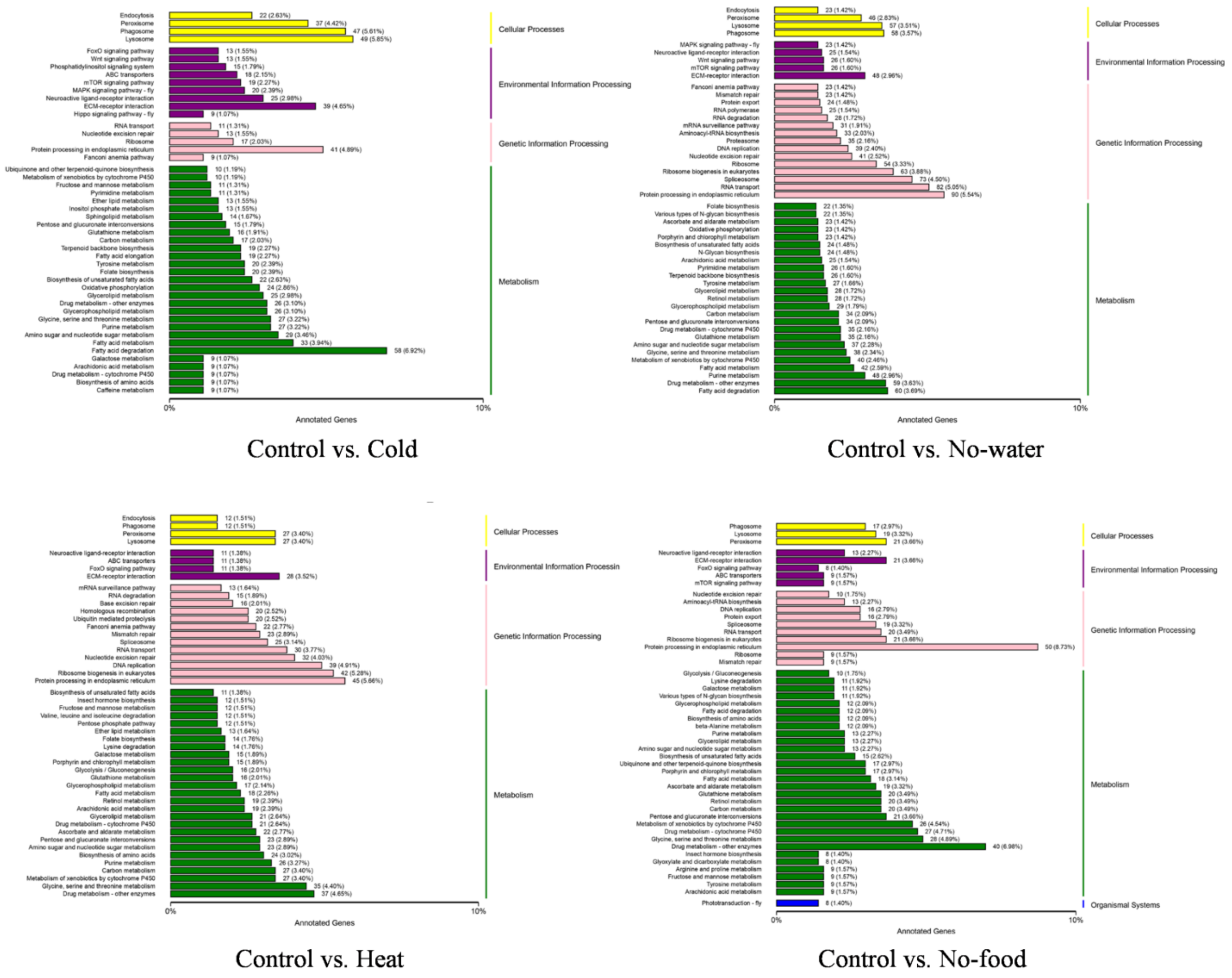


**Figure S2.** The KEGG pathway classification of DEGs in the brain samples of S. *frugiperda* under four different stress conditions.

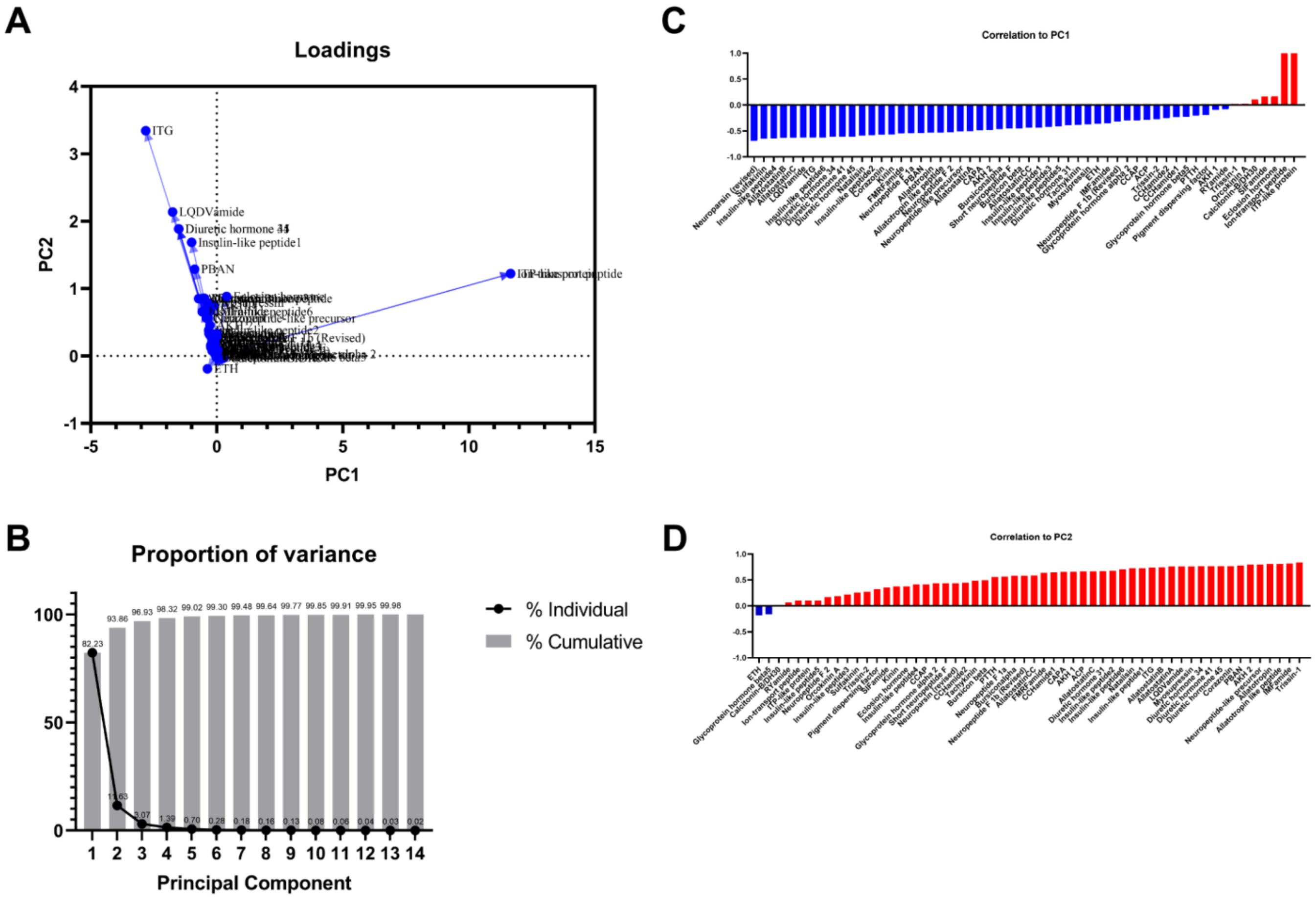


**Figure S3.** Projection of the variables (genes) on the correlation graph is shown in panel A. Panel B presents the proportion of variance (black line) explained by successive PCs together with cumulative values of following PCs - barplot, the gene correlations to PC1 are presented in panel C and to PC2 in panel D, with blue columns denoting negative correlation and the red columns postive.

**Table S1.** The absolute expression (mean±SEM) of top 50 up- and downregulated *DEGs* in the brain samples of *S. frugiperda* under "cold stress".

| Gene ID | Gene description | Control | Cold stress |
|---|---|---|---|
| **LOC118276202** | cytochrome c oxidase assembly factor 5 | 7.05±0.45 | 0±0 |
| **LOC118277167** | uncharacterized protein | 5.52±1.20 | 0±0 |
| **LOC118281031** | cholesterol 7-desaturase nvd | 1.14±0.18 | 0±0 |
| **LOC118277557** | uncharacterized protein LOC118277557 | 5.21±1.45 | 0.05±0.08 |
| **LOC118282292** | inducible metalloproteinase inhibitor protein-like | 29.1±2.1 | 0.61±0.33 |
| **LOC118270577** | cytochrome P450 4C1-like | 3.69±0.75 | 0.09±0.17 |
| **LOC118276806** | uncharacterized LOC118276806 | 47.4±3.8 | 1.63±0.77 |
| **LOC118275600** | fibroin heavy chain-like | 300±10 | 6.04±1.53 |
| **LOC118271791** | protein NDNF-like | 5.09±0.71 | 0.28±0.32 |
| **LOC118263083** | cytochrome P450 6k1-like | 1.07±0.26 | 0.03±0.11 |
| **LOC118281189** | keratin, type I cytoskeletal 10-like | 19.4±1.2 | 0.19±0.28 |
| **LOC118275334** | uncharacterized PE-PGRS family protein PE_PGRS54 | 40.2±2.7 | 2.23±0.78 |
| **LOC118282322** | uncharacterized LOC118274922 | 2.00±0.44 | 0.17±0.23 |
| **LOC118274922** | cytochrome P450 4C1-like | 5.53±0.79 | 0.42±0.21 |
| **LOC118270313** | aldo-keto reductase AKR2E4-like | 4.39±1.10 | 0.31±0.29 |
| **LOC118279827** | S-phase kinase-associated protein 2-like | 8.09±0.89 | 0.68±0.50 |
| **LOC118268215** | uncharacterized LOC118268215 | 0.88±0.22 | 0.06±0.08 |
| **LOC118268215** | uncharacterized LOC118278672 | 3.01±0.43 | 0.25±0.26 |
| **LOC118278672** | sorbitol dehydrogenase-like | 4.58±0.80 | 0.42±0.24 |
| **LOC118274356** | uncharacterized LOC118274096 | 4.07±0.74 | 0.37±0.26 |
| **LOC118274096** | uncharacterized LOC118278392 | 60.3±2.7 | 5.69±1.12 |
| **LOC118278392** | balbiani ring protein 3-like | 0.56±0.23 | 0.05±0.07 |
| **LOC118272505** | senecionine N-oxygenase | 0.49±0.18 | 0.05±0.1 |
| **LOC118264690** | cytochrome P450 6j1 | 4.29±0.70 | 0.44±0.19 |
| **LOC118265346** | tRNA (cytosine (38)-C (5))-methyltransferase-likeTRNA | 2.93±0.29 | 0.32±0.28 |
| **LOC118266484** | cuticle protein 3-like | 0±0 | 41.4±3.0 |
| **LOC118261791** | cuticle protein 19-like | 0±0 | 18.1±1.8 |
| **LOC118280439** | putative fatty acyl-CoA reductase CG5065 | 0.17±0.23 | 105±4 |
| **LOC118265850** | pupal cuticle protein 27-like | 0±0 | 3.72±0.31 |
| **LOC118266107** | pupal cuticle protein 27-like | 0±0 | 2.18±0.32 |
| **LOC118264729** | ecdysone oxidase | 0.32±0.22 | 57.5±2.7 |
| **LOC118279248** | juvenile hormone epoxide hydrolase-like | 0±0 | 0.84±0.22 |
| **LOC118282152** | organic cation transporter protein | 0.21±0.14 | 29.8±2.2 |
| **LOC118274135** | cuticle protein 8-like | 2.16±0.72 | 413±5 |
| **LOC118274039** | zinc transporter 2 | 4.06±0.77 | 487±9 |
| **LOC118274155** | mucin-5AC | 0.00±0.03 | 0.97±0.52 |
| **LOC118279356** | chitin deacetylase 8-like | 0.01±0.072 | 2.82±0.67 |
| **LOC118271325** | uncharacterized LOC118271325 | 0.30±0.20 | 30.1±1.5 |
| **LOC118261742** | histidine-rich glycoprotein-like | 0.10±0.15 | 14.0±1.4 |
| **LOC118272743** | cuticle protein 63-like | 2.44±0.79 | 266±4 |
| **LOC118266033** | farnesyl pyrophosphate synthase 1-like | 0.34±0.25 | 32.5±2.1 |
| **LOC118277982** | larval cuticle protein A2B-like | 1.43±0.67 | 148±1 |
| **LOC118265488** | histidine-rich glycoprotein-like | 0.03±0.12 | 3.44±0.61 |
| **LOC118271331** | uncharacterized LOC118271331 | 5.22±1.33 | 1570±12 |
| **LOC118274766** | alpha-tocopherol transfer protein-like | 0.60±0.20 | 37.6±1.2 |
| **LOC118279156** | la-related protein 6La | 6.91±1.22 | 482±5 |

| | | | |
|---|---|---|---|
| **LOC118277977** | cuticle protein 19.8 | 1.01±0.64 | 119±3 |
| **LOC118275529** | uncharacterized LOC118275529 | 2.37±0.56 | 149±5 |
| **LOC118275071** | uncharacterized LOC118275071 | 0.23±0.20 | 15.8±1.4 |
| **LOC118275532** | lopap-like | 0.17±0.20 | 13.0±1.2 |

The absolute expression scalar uses three technically repeated FPKM values, and the independent sample t test was used in SPSS22.0 to calculate mean±SEM.

**Table S2**. The absolute expression (mean±SEM) of top 50 up- and downregulated *DEGs* in the brain samples of *S. frugiperda* under "heat stress".

| Gene ID | Gene description | Control | Heat stress |
|---|---|---|---|
| **LOC118280746** | protein lethal (2) essential for life-like | 0.27±0.23 | 2006±10 |
| **LOC118267423** | uncharacterized LOC118267423 | 0.03±0.07 | 195±3 |
| **LOC118267445** | uncharacterized LOC118267445 | 0.04±0.07 | 222±3 |
| **LOC118270841** | uncharacterized LOC118270841 | 0.11±0.13 | 228±4 |
| **LOC118280753** | protein lethal (2) essential for life-like | 0.08±0.14 | 194±5 |
| **LOC118277229** | protein lethal (2) essential for life-like | 0.06±0.13 | 463±7 |
| **LOC118270842** | uncharacterized LOC118270842 | 0±0 | 49.3±2.1 |
| **LOC118262918** | ichor | 0.12±0.10 | 85.7±2.5 |
| **LOC118270676** | uncharacterized LOC118270676 | 0±0 | 14.6±0.8 |
| **LOC118270054** | uncharacterized LOC118270054 | 0.10±0.13 | 56.0±1.3 |
| **LOC118275417** | uncharacterized LOC118275417 | 1.37±0.18 | 557±7 |
| **LOC118280707** | heat shock protein 70 A1 | 0.33±0.16 | 134±3 |
| **LOC118270675** | uncharacterized LOC118270675 | 0.34±0.26 | 170±3 |
| **LOC118280660** | heat shock protein 68 | 1.00±0.25 | 394±6 |
| **LOC118270503** | uncharacterized LOC118270503 | 0.04±0.12 | 17.2±0.4 |
| **LOC118278157** | heat shock protein Hsp-12.2-like | 1.72±0.27 | 504±5 |
| **LOC118280747** | protein lethal (2) essential for life | 0.42±0.28 | 159±4 |
| **LOC118280771** | protein lethal (2) essential for life | 0.93±0.12 | 271±4 |
| **LOC118272531** | cuticlin-4 | 0.15±0.15 | 45.8±1.4 |
| **LOC118280409** | trypsin-like | 0±0 | 7.61±0.84 |
| **LOC118273355** | uncharacterized LOC118273355 | 0.18±0.10 | 47.9±1.3 |
| **LOC118267654** | uncharacterized LOC118267654 | 0.46±0.11 | 86.3±2.5 |
| **LOC118280709** | uncharacterized LOC118280709 | 0.03±0.09 | 10.3±1.1 |
| **LOC118280708** | heat shock protein 68-like | 0.69±0.35 | 285±5 |
| **LOC118280663** | major heat shock 70 kDa protein Ba | 0.82±0.42 | 472±6 |
| **LOC118282125** | chitin deacetylase 1 | 69.2±2.3 | 0±0 |
| **LOC118268104** | cytochrome b5-related protein | 13.8±1.1 | 0.08±0.08 |
| **LOC118263791** | uncharacterized oxidoreductase YrbE-like | 2.62±0.63 | 0±0 |
| **Spodoptera_frugiperda_ newGene_380** | diapausin precursor | 384±6 | 5.15±0.89 |
| **LOC118265576** | spore coat protein T-like | 2.09±0.43 | 0±0 |
| **LOC118269312** | putative inorganic phosphate cotransporter | 1.11±0.25 | 0.02±0.07 |
| **LOC118274356** | sorbitol dehydrogenase-like | 4.07±0.74 | 0.09±0.15 |
| **LOC118281189** | cytochrome P450 307a1 | 19.4±1.2 | 0.65±0.35 |
| **LOC118274074** | ribose-phosphate pyrophosphokinase 2-like | 9.11±0.91 | 0.37±0.25 |
| **LOC118279078** | glucose dehydrogenase [FAD, quinone]-like | 5.38±0.68 | 0.22±0.18 |
| **LOC118264370** | ecdysone oxidase-like | 1.04±0.34 | 0.02±0.09 |
| **LOC118268452** | cytochrome b5-related protein-like | 6.81±0.91 | 0.05±0.13 |
| **LOC118276416** | glucose dehydrogenase [FAD, quinone]-like | 0.94±0.26 | 0.04±0.09 |
| **LOC118264063** | 15-hydroxyprostaglandin dehydrogenase [NAD (+)]-like | 3.64±0.73 | 0.19±0.1 |
| **LOC118265511** | aminoacylase-1 | 2.18±0.44 | 0.12±0.11 |
| **LOC118273698** | androgen-dependent TFPI-regulating protein-like | 3.97±0.64 | 0.2±0.21 |
| **LOC118280769** | vitellogenin | 86.9±3.0 | 5.87±0.87 |
| **LOC118266066** | TATA-binding protein-associated factor 2N-like | 24.7±1.8 | 1.43±0.5 |

| | | | |
|---|---|---|---|
| **LOC118264969** | chromatin assembly factor 1 subunit B | 4.67±0.56 | 0.32±0.21 |
| **LOC118272704** | uncharacterized LOC118272704 | 5.08±0.59 | 0.32±0.23 |
| **LOC118266683** | argininosuccinate lyase | 1.39±0.39 | 0.08±0.05 |
| **LOC118265285** | chromatin assembly factor 1 subunit B-like | 5.71±0.60 | 0.41±0.20 |
| **LOC118267238** | DNA polymerase delta catalytic subunit | 3.78±0.25 | 0.30±0.10 |
| **LOC118266621** | fidgetin-like protein 1 | 1.35±0.21 | 0.09±0.12 |
| **LOC118267757** | uncharacterized LOC118267757 | 6.69±0.45 | 0.49±0.26 |

The absolute expression scalar uses three technically repeated FPKM values, and the independent sample t test was used in SPSS22.0 to calculate mean±SEM.

**Table S3.** The absolute expression (mean±SEM) of top 50 up- and downregulated *DEGs* in the brain samples of *S. frugiperda* under "no-water stress".

| Gene ID | Gene description | Control | No-water stress |
|---|---|---|---|
| **LOC118281031** | cholesterol 7-desaturase-like | 1.14±0.07 | 0±0 |
| **LOC118264791** | glucose dehydrogenase [FAD, quinone]-like | 2.12±0.27 | 0.02±0.01 |
| **LOC118265091** | uncharacterized protein LOC118265091 | 0.97±0.25 | 0.01±0.01 |
| **LOC118264370** | glucose dehydrogenase [FAD, quinone]-like | 1.04±0.25 | 0.01±0.01 |
| **LOC118265142** | phospholipase A1-like | 14.86±3.79 | 0.20±0.11 |
| **LOC118280769** | vitellogenin-like | 86.9±19.0 | 1.75±0.62 |
| **LOC118264141** | solute carrier family 23 member 2-like isoform X1 | 4.34±0.90 | 0.09±0.02 |
| **LOC118266985** | uncharacterized protein LOC118266985 isoform X1 | 25.2±2.0 | 0.58±0.15 |
| **LOC118279815** | probable nuclear hormone receptor HR3 isoform X1 | 65.0±7.0 | 1.48±0.71 |
| **LOC118267572** | neurogenic locus protein delta-like isoform X1 | 6.55±0.78 | 0.15±0.04 |
| **LOC118272271** | arylphorin subunit alpha-like | 15.1±12.2 | 0.04±0.03 |
| **LOC118281189** | cytochrome P450 307a1-like | 19.4±2.9 | 0.39±0.17 |
| **LOC118281574** | uncharacterized protein LOC118281574 | 3.28±0.50 | 0.07±0.05 |
| **LOC118271791** | uncharacterized protein LOC118271791 | 5.09±1.07 | 0.12±0.04 |
| **LOC118271700** | arylphorin subunit alpha-like | 28.7±22.7 | 0.23±0.10 |
| **LOC118279845** | probable nuclear hormone receptor HR3 isoform X6 | 47.9±1.8 | 1.11±0.68 |
| **Spodopterafrugiperdanew Gene2653** | probable rRNA-processing protein EBP2 homolog | 10.3±1.5 | 0.33±0.04 |
| **LOC118275600** | fibroin heavy chain-like | 300±229 | 1.29±1.24 |
| **LOC118265961** | glycine-rich cell wall structural protein-like | 555±79 | 16.4±11.0 |
| **LOC118270723** | uncharacterized protein LOC118270723 | 39.2±3.2 | 1.32±0.78 |
| **LOC118278979** | uncharacterized protein LOC118278979 | 3.83±0.70 | 0.11±0.07 |
| **LOC118270699** | uncharacterized protein LOC118270699 | 19.2±1.7 | 0.63±0.46 |
| **LOC118266599** | uncharacterized protein LOC118266599 | 23.6±2.4 | 0.83±0.50 |
| **LOC118277508** | serine protease inhibitor dipetalogastin-like isoform X1 | 9.15±1.46 | 0.32±0.17 |
| **LOC118265700** | uncharacterized protein LOC111361849 | 71.0±5.5 | 1.52±1.33 |
| | | | |
| **LOC118266484** | cuticle protein 3-like | 0±0 | 43.8±21.7 |
| **LOC118280439** | putative fatty acyl-CoA reductase CG5065 | 0.17±0.11 | 49.1±13.4 |
| **LOC118264729** | glucose dehydrogenase [FAD, quinone]-like | 0.32±0.10 | 36.0±6.0 |
| **LOC118277985** | larval cuticle protein A2B-like | 0.69±0.46 | 59.3±25.9 |
| **LOC118282152** | organic cation transporter protein-like | 0.21±0.04 | 11.4±3.2 |
| **LOC118277982** | larval cuticle protein A2B-like | 1.43±0.96 | 93.3±26.1 |
| **LOC118261951** | inducible metalloproteinase inhibitor protein-like | 0.04±0.02 | 2.71±0.66 |
| **LOC118274039** | zinc transporter 2-like isoform X1 | 4.06±1.26 | 181±33 |
| **LOC118272743** | cuticle protein 63-like | 2.44±1.32 | 134±23 |
| **LOC118272878** | RNA-binding protein 33-like | 0.04±0.02 | 7.11±3.11 |
| **LOC118263357** | cytochrome P450 4g15-like | 19.5±7.5 | 812±111 |
| **LOC118272702** | uncharacterized protein LOC118272702 | 0.12±0.05 | 5.22±2.46 |

| | | | |
|---|---|---|---|
| **Spodoptera_frugiperda_ newGene_637** | -- | 0.29±0.16 | 14.8±5.0 |
| **LOC118264484** | uncharacterized protein LOC118264484 | 0±0 | 2.37±1.88 |
| **LOC118275532** | lopap-like | 0.17±0.08 | 6.83±0.91 |
| **LOC118261945** | cuticle protein 8-like | 0.33±0.10 | 15.2±6.6 |
| **LOC118270763** | cytochrome P450 4c21-like | 0.96±0.51 | 39.4±9.7 |
| **LOC118281584** | histidine-rich glycoprotein-like | 0.95±0.14 | 48.4±26.7 |
| **LOC118274766** | alpha-tocopherol transfer protein-like | 0.60±0.08 | 19.8±6.0 |
| **LOC118278128** | uncharacterized protein LOC118278128 | 0.07±0.04 | 2.97±1.07 |
| **LOC118272316** | xylose isomerase-like | 0.27±0.10 | 8.50±1.40 |
| **LOC118281597** | histidine-rich glycoprotein-like | 0.27±0.10 | 77.5±38.0 |
| **LOC118276439** | uncharacterized protein LOC118276439 | 0.02±0.01 | 0.70±0.07 |
| **LOC118263590** | trypsin 3A1-like | 0.42±0.09 | 13.8±4.6 |
| **LOC118266606** | uncharacterized protein LOC118266606 | 4.72±0.97 | 140±48 |
| **LOC118274135** | cuticle protein 8-like | 2.16±1.09 | 153±12 |

The absolute expression scalar uses three technically repeated FPKM values, and the independent sample t test was used in SPSS22.0 to calculate mean±SEM.

**Table S4.** The absolute expression (mean±SEM) of top 50 up- and downregulated *DEGs* in the brain samples of *S. frugiperda* under "no-food stress".

| Gene ID | Gene description | Control | No-food stress |
|---|---|---|---|
| **LOC118280769** | vitellogenin-like | 86.9±19.0 | 0.91±0.11 |
| **LOC118263791** | LOW QUALITY PROTEIN: uncharacterized oxidoreductase YrbE-like | 2.62±0.83 | 0±0 |
| **LOC118280768** | uncharacterized protein LOC118280768 | 59.4±14.6 | 0.90±0.05 |
| **LOC118279815** | probable nuclear hormone receptor HR3 isoform X1 | 65.0±7.0 | 0.9±0.39 |
| **LOC118280767** | vitellogenin-like | 36.3±9.4 | 0.70±0.02 |
| **LOC118267572** | neurogenic locus protein delta-like isoform X1 | 6.55±0.78 | 0.18±0.04 |
| **LOC118266985** | uncharacterized protein LOC118266985 isoform X1 | 25.2±2.0 | 0.48±0.29 |
| **LOC118274075** | beta-ureidopropionase-like | 13.0±1.8 | 0.38±0.14 |
| **LOC118281189** | cytochrome P450 307a1-like | 19.4±2.9 | 0.81±0.16 |
| **LOC118271791** | uncharacterized protein LOC118271791 | 5.09±1.07 | 0.14±0.08 |
| **LOC118269843** | uncharacterized protein LOC118269843 | 14.8±4.6 | 0.43±0.10 |
| **LOC118266599** | uncharacterized protein LOC118266599 | 23.6±2.4 | 1.08±0.26 |
| **LOC118279845** | probable nuclear hormone receptor HR3 isoform X6 | 47.9±1.8 | 0.67±0.34 |
| **LOC118267757** | uncharacterized protein LOC118267757 | 6.69±0.44 | 0.33±0.08 |
| **LOC118274305** | D-amino-acid oxidase-like | 3.83±0.42 | 0.11±0.07 |
| **LOC118278744** | hemolin-like | 229±48 | 10.3±5.4 |
| **LOC118268104** | cytochrome b5-related protein-like | 13.8±2.7 | 0.73±0.21 |
| **LOC118269804** | lipid storage droplets surface-binding protein 1-like isoform X1 | 4.73±0.65 | 0.20±0.10 |
| **LOC118275940** | UDP-glucuronosyltransferase 1-2-like | 4.44±1.21 | 0.19±0.07 |
| **LOC118268452** | cytochrome b5-related protein-like | 6.81±1.75 | 0.36±0.07 |
| **LOC118271974** | carboxypeptidase B-like | 50.9±9.41 | 3.52±0.72 |
| **LOC118263297** | glycine N-methyltransferase-like | 6.13±0.69 | 0.48±0.07 |
| **LOC118276924** | ommochrome-binding protein-like | 2.76±0.30 | 0.19±0.05 |
| **LOC118268856** | glycine cleavage system H protein-like isoform X1 | 7.65±1.56 | 0.53±0.09 |
| **LOC118282322** | uncharacterized PE-PGRS family protein PE_PGRS54-like isoform X1 | 2.00±0.41 | 0.13±0.05 |
| **LOC118267373** | LOW QUALITY PROTEIN: probable 4-coumarate--CoA ligase 1 | 1.91±0.20 | 37.88±9.66 |
| **Spodoptera_frugiperda_ newGene_364** | -- | 6.88±2.28 | 130±5 |
| **LOC118261951** | inducible metalloproteinase inhibitor protein-like | 0.04±0.02 | 1.54±0.26 |
| **LOC118274094** | cytochrome P450 6B6-like | 0.13±0.05 | 2.88±0.73 |
| **LOC118280239** | dehydrogenase-like | 0.21±0.08 | 2.74±0.45 |
| **LOC118279537** | aromatic-L-amino-acid | 0.11±0.03 | 1.35±0.29 |

| | | | |
|---|---|---|---|
| | decarboxylase-like | | |
| **LOC118263839** | balbiani ring protein 3-like isoform X1 | 0.15±0 | 1.37±0.21 |
| **LOC118264056** | cytochrome P450 9e2-like | 0.92±0.22 | 7.75±0.76 |
| **LOC118279835** | cytochrome P450 6B7-like | 2.26±0.44 | 19.0±5.9 |
| **LOC118280439** | putative fatty acyl-CoA reductase CG5065 | 0.17±0.11 | 2.88±1.67 |
| **LOC118272314** | LOW QUALITY PROTEIN: glutaminase liver isoform, mitochondrial-like | 14.4±2.6 | 93.9±13.9 |
| **LOC118279736** | cytochrome P450 6B7-like | 3.37±0.71 | 24.2±6.3 |
| **LOC118266455** | allergen Tha p 1-like | 499±66 | 3123±559 |
| **LOC118279687** | histidine decarboxylase-like isoform X1 | 0.16±0.06 | 1.37±0.33 |
| **LOC118277165** | uncharacterized protein LOC118277165 | 13.3±2.9 | 95.4±31.1 |
| **LOC118274574** | luciferin 4-monooxygenase-like | 0.34±0.07 | 2.33±0.49 |
| **LOC118264989** | uncharacterized protein LOC118264989 | 3.38±0.34 | 19.1±3.2 |
| **LOC118277902** | UDP-glucuronosyltransferase 2B1-like | 3.30±1.09 | 21.5±4.3 |
| **LOC118280743** | protein lethal(2)essential for life-like | 26.7±5.4 | 164±42 |
| **LOC118282442** | inducible metalloproteinase inhibitor protein-like | 1.70±0.49 | 13.1±5.4 |
| **LOC118275934** | interaptin-like isoform X1 | 0.68±0.09 | 3.77±0.66 |
| **LOC118272200** | IST1-like protein | 4.33±1.21 | 24.90±2.75 |
| **LOC118279357** | UDP-glucuronosyltransferase 2B7-like | 1.28±0.20 | 6.34±0.45 |
| **LOC118274766** | alpha-tocopherol transfer protein-like | 0.60±0.08 | 3.19±0.20 |
| **LOC118263357** | cytochrome P450 4g15-like | 19.5±7.5 | 200±65 |
| **LOC118278440** | modular serine protease-like | 0.53±0.14 | 3.60±1.63 |

The absolute expression scalar uses three technically repeated FPKM values, and the independent sample t test was used in SPSS22.0 to calculate mean±SEM.
